# Tunable light and drug induced depletion of target proteins

**DOI:** 10.1038/s41467-019-14160-8

**Published:** 2020-01-16

**Authors:** Wen Deng, Jack A. Bates, Hai Wei, Michael D. Bartoschek, Barbara Conradt, Heinrich Leonhardt

**Affiliations:** Department of Biology II, Ludwig-Maximilians-Universität München, Munich, Germany

**Keywords:** Biological techniques, Cell biology, Developmental biology

## Abstract

Biological processes in development and disease are controlled by the abundance, localization and modification of cellular proteins. We have developed versatile tools based on recombinant E3 ubiquitin ligases that are controlled by light or drug induced heterodimerization for nanobody or DARPin targeted depletion of endogenous proteins in cells and organisms. We use this rapid, tunable and reversible protein depletion for functional studies of essential proteins like PCNA in DNA repair and to investigate the role of CED-3 in apoptosis during *Caenorhabditis elegans* development. These independent tools can be combined for spatial and temporal depletion of different sets of proteins, can help to distinguish immediate cellular responses from long-term adaptation effects and can facilitate the exploration of complex networks.

## Abstract (web summary)

Methods to control protein degradation usually require prior genetic engineering of each specific protein of interest. Here the authors present a toolbox for targeted depletion of endogenous proteins in cells and organisms, which is based on light- and drug-controlled recombinant E3 ubiquitin ligases.

## Introduction

The cellular abundance of proteins is determined by transcription, translation and degradation, each of which may be targeted for functional studies. Genetic methods, including the recent variants of gene editing, are precise and versatile but not dynamic, which may over time provoke cellular adaptations and complicate functional analyses^1^. In contrast, the transient and tunable methods targeting specific mRNAs, mostly by RNA interference, are less efficient and not suited for stable proteins with little natural turnover^2^ or maternally provided proteins. Therefore, current protein knockdown techniques, directly target proteins of interest (POIs) with the cellular ubiquitin-proteasome system (UPS), mainly using two strategies. Degradation domains or degrons, which are protein fragments mediating degradation of protein, were genetically fused to POIs to control their stabilities^3–12^. Alternatively, the mammalian F-box protein, which is a component of the SCF E3 ubiquitin ligase complex, was directed with a GFP binding vhh4 nanobody^13^ to degrade fluorescent fusion proteins^14^. Recently, the auxin-induced protein degradation system was generalized by combining the auxin-induced degron (AID) and GFP binding nanobody to degrade GFP fusion protein controllably^15^. In these cases, the stability of POIs can be dynamically controlled with small molecules (like auxin or Shield-1), but targeted destruction requires prior genetic engineering to render POIs susceptible.

Targeting endogenous proteins has been demonstrated with the Trim-Away method^16,17^, which is based on the TRIM21 E3 ubiquitin ligase. TRIM21 binds antibodies and ubiquitinates their bound antigens marking them for proteasomal degradation. Here the need for genetic manipulations is replaced by physical microinjection of purified antibodies, which requires specialized equipment and sustained depletion requires repeated injections. Alternatively, ubiquitin ligases and POIs may be bound and connected with bi-functional small molecules named proteolysis targeting chimera (PROTAC)^18^, which bypasses the need for genetic engineering but requires selection and chemical engineering of cell permeable small molecules with dual binding specificity.

The goal of this study is to develop versatile toolsets for efficient and controlled depletion of tagged as well as untagged endogenous target proteins in cells and organisms. To study complex cellular systems it is desirable to have inducible tools for defined starting points and reversibility to monitor the response to transient interference. Any system should be tunable to gradually control protein levels, to probe stoichiometric requirements and to define rate-limiting levels. To address redundancies and interdependencies it would be helpful to target sets of proteins in freely selectable temporal and spatial combinations. Here, we present light and drug-controlled depletion tools that can be used in combination to control cellular levels of two or more sets of proteins.

## Results

### Screening E3 ubiquitin ligases for protein depletion

For ubiquitination, proteins are recognized and bound by the substrate binding part of specific E3 ubiquitin ligases, which ligate ubiquitin to the substrate proteins via their catalytic domain. As a general strategy we replaced the native substrate binding domain of E3 ubiquitin ligases with recombinant binding modules to direct the ubiquitin ligase activity to selected target proteins and mark them for depletion. For optimal efficiency we first tested and compared the catalytic activity of different types of E3 ubiquitin ligase domains, including F-box, BTB, RING, and HECT domains. To be able to directly monitor depletion efficiency we chose a green fluorescent fusion protein (GFP-CXXC4, GFP fused to the N-terminus of CXXC-type zinc finger protein 4) as a test target and fused the ligase domains with the GFP binding nanobody GBP1 (GFP binding protein 1)^13^. Ligase fusions were introduced into HeLa cells stably expressing the target (GFP-CXXC4) and fluorescence was monitored by microscopy or FACS. The side-by-side comparison of ubiquitin ligase constructs showed varying reductions in fluorescence intensities with the RING domain of LNX1 protein (RING^Lnx1^) functioning most effectively and reaching depletion efficiencies of more than 95% (Supplementary Fig. 1a, b). Similarly, a second target protein (GFP-PCNA, GFP fused to Proliferating Cell Nuclear Antigen protein) was also most efficiently depleted with the RING^Lnx1^ (Supplementary Fig. 1c). Based on sequence alignments and the crystal structure of the TRAF6 RING domain^19^, we tried to further shorten the RING^Lnx1^ domain; however, the shorter construct (RING^Lnx1 sh^) failed to degrade the target (GFP-PCNA), indicating that the RING^Lnx1^ domain is optimal for protein depletion (Supplementary Fig. 1d, e). Compared to other tested domains, RING^Lnx1^ was the smallest and most efficient (Supplementary Fig. 1f); therefore, we chose this E3 ubiquitin ligase domain for further tool development.

### Development of a light induced protein depletion tool

**Fig. 1:**
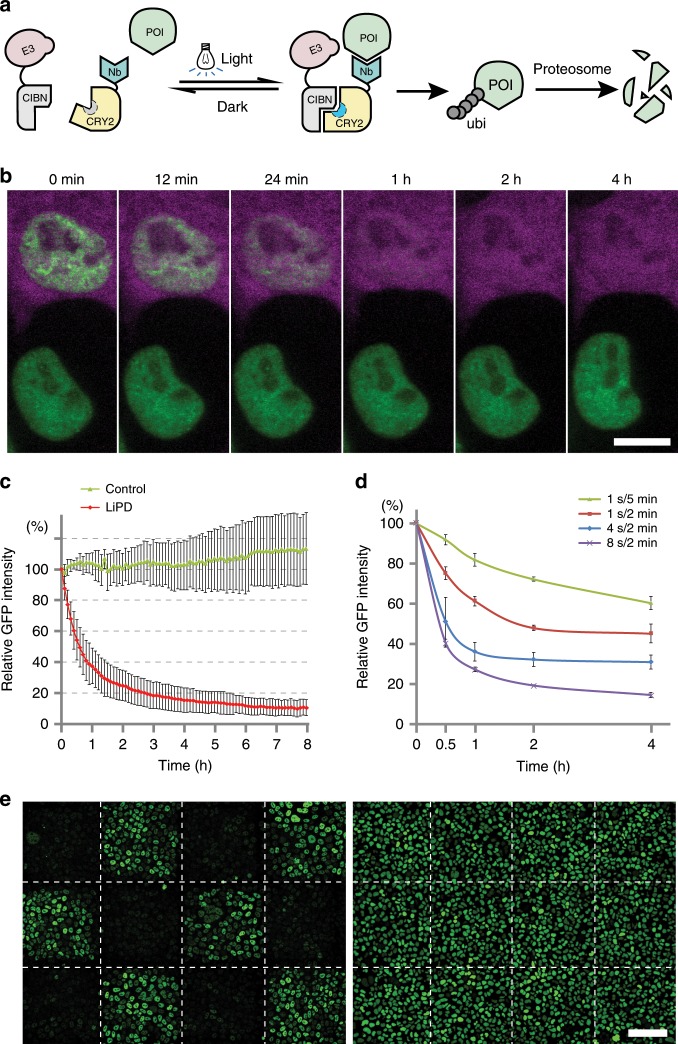
Light induced protein depletion. **a** Schematic representation of the light induced protein depletion tool. Short light pulses trigger a conformational switch in Cryptochrome 2 (CRY2) and thus allow heterodimerization with CIBN (cryptochrome-interacting basic-helix-loop-helix 1 N-terminus). The light induced heterodimerization brings together the nanobody bound protein of interest (POI) and the E3 ubiquitin ligase (E3) causing ubiquitination and depletion of the target protein. E3 RING domain of E3 ubiquitin ligase, Nb Nanobody, POI protein of interest, ubi ubiquitin. **b** Light treatment induced rapid depletion of the target protein (in green) in cells containing the LiPD system (magenta) but not in control cells. Scale bar is 10 µm. **c** Depletion kinetics of GFP-CXXC4 with the LiPD system. For LiPD cells, *n* = 27; for control cells, *n* = 16. Intensity values are shown as mean ± SD. **d** Tunable regulation of GFP-CXXC4 abundance across a whole-cell population with diverse illuminating programs using an LED lightbox. Biological triplicates were measured; Intensity values are shown as mean ± SD. **e** Spatial control of protein depletion with the LiPD system. Stable LiPD/GFP-CXXC4 cells were exposed to a chess board pattern of light, leading to depletion of GFP-CXXC4 in the illuminated squares (left). GFP-CXXC4 cells without LiPD system were treated using the same lightening program to exclude loss of fluorescence due to photobleaching (right). Scale bar is 100 µm.

To be able to control and finetune the targeted ubiquitination, we separated target binding from catalytic activity and fused them to light sensitive heterodimerization modules. Upon short exposure to blue light the conformation of the light responsive protein changes and thereby triggers heterodimer formation. This light induced heterodimerization brings together the target binding nanobody and the ubiquitin ligase domain to mark proteins of interest (POIs) for degradation (Fig. 1a). For the implementation of light induced protein depletion (LiPD), we tested two protein pairs, iLID/SspB^20^ and CIBN/CRY2^21^, which dimerize upon blue light triggered conformational change. As the kinetics of light induced dimerization (LID) and especially the dissociation rate might be critical for specific applications, we measured these parameters with live cell microscopy. In brief, one half (iLID or CIBN) was anchored at a defined subcellular site, the cell membrane or focal nuclear replication sites via a GFP binding nanobody, and the enrichment of the corresponding other half (SspB or CRY2, respectively) was monitored over time (Supplementary Fig. 2). In comparison, the CIBN/CRY2 pair seemed more efficient in the recruitment of proteins at the cell membrane with dissociation half times of about 5 min (Supplementary Fig. 2a, b). We further tested the CIBN/CRY2 dimerization pair in the nucleus using replication protein PCNA as anchoring structures (Supplementary Fig. 2c). The fine punctate pattern of these PCNA-labeled replication foci clearly shows the rapid recruitment of the PHR domain of CRY2 (PHR^cry2^) within a few seconds after light exposure (Supplementary Fig. 2d and Movie 1). Quantitative analysis of several cells showed a slow dissociation of PHR^cry2^-mCh with a half time of about 6 min (Supplementary Fig. 2e). While the short-lived iLID/SspB dimer might be of advantage for some applications, we chose the more efficient and stable CIBN/CRY2 dimerization pair for subsequent protein depletion experiments.

We then tested different configurations and combinations of the CIBN/CRY2 dimerization pair and the protein targeting/depletion modules to optimize the LiPD system. We found that the GBP1-PHR^cry2^/RING^Lnx1^-CIBN combination showed the best depletion of GFP-PCNA upon light induction (Supplementary Fig. 3a, b). Of the tested GFP binding nanobodies, GBP1 performed best in depletion of GFP-PCNA (Supplementary Fig. 3c) and was chosen for subsequent experiments. Transient co-expression of these optimized components enabled rapid depletion of GFP-PCNA after light induction and caused a GFP signal decrease to about 20% of the initial intensities within 4 h (Supplementary Fig. 3d, e and Movie 2).

To enhance the efficiency of the LiPD, we aimed for a stable and balanced stoichiometry between the two components by expressing them with similar promoters (mouse and rat EF1a promoters) on a single vector, which can easily be integrated into the genome via the piggyBac transposon (Supplementary Fig. 3f). We inserted the LiPD system into the GFP-CXXC4-expressing cell line. Without light induction, stable expression of the LiPD system caused only a slight reduction (about 3%) of the fluorescence, which might be due to ambient light during cell culture and spontaneous heterodimer formation (Supplementary Fig. 3g, h). After light induction, GFP-CXXC4 levels decreased quickly, but not in cells without the LiPD system (Fig. 1b, and Supplementary Movie 3). Quantitative imaging showed that depletion of the GFP fusion protein was rapid and could be observed immediately after light induction, with a half depletion time of about 30 min (Fig. 1c). GFP-CXXC4 was depleted to a level below 10% in cells with the LiPD (Fig. 1c), indicating a fast and efficient regulation of protein abundance in living cells. To apply this LiPD technology to other cellular targets, we generated a LiPD cell line and inserted GFP at the endogenous *CENPA* locus by a CRISPR/Cas9 mediated gene editing and recombineering technology^22^ (Supplementary Fig. 4a, b). Also in this case, live cell imaging showed a fast and almost complete depletion of GFP-CENPA after light illumination (Supplementary Fig. 4c, d and Movie 4). Since the GFP knock-in was heterozygotic, only half of the cellular CENPA was GFP- tagged and depleted after light induction, thus no mitotic defect was observed during the whole imaging period.

To control proteostasis across large cell populations, we constructed a simple LED lightbox, which can be programmed to expose cells in culture dishes to 470 nm light at defined time intervals (Supplementary Fig. 5a). The lightbox was first tested by monitoring the induction of dimerization between the LID protein pairs (Supplementary Fig. 5b, c) and then the depletion of GFP-CENPA protein (Supplementary Fig. 5d, e). To test whether the GFP fusion protein levels can be tuned and continuously regulated with light, the LiPD/GFP-CXXC4 cell line was treated with different illumination programs (Supplementary Fig. 5f), and fluorescence intensities of the whole-cell populations were measured with a fluorescence reader at different time points. With these illumination programs, the GFP fusion protein was depleted with different kinetics down to amounts ranging from 20 to 70% of the initial values, indicating a dose-dependent regulation of proteostasis across the cell population (Fig. 1d).

Besides this dose and time control, the physical nature of the LiPD system also offers spatial control. To explore this option, light induction was applied in a chess board pattern to the GFP-CXXC4 cells with stably integrated LiPD. This patterned illumination resulted in a local depletion of the targeted GFP fusion protein, which demonstrates efficient spatial control of protein levels and allows the direct side-by-side comparison with neighboring non-depleted cells (Fig. 1e).

### Development of drug-induced protein depletion

**Fig. 2:**
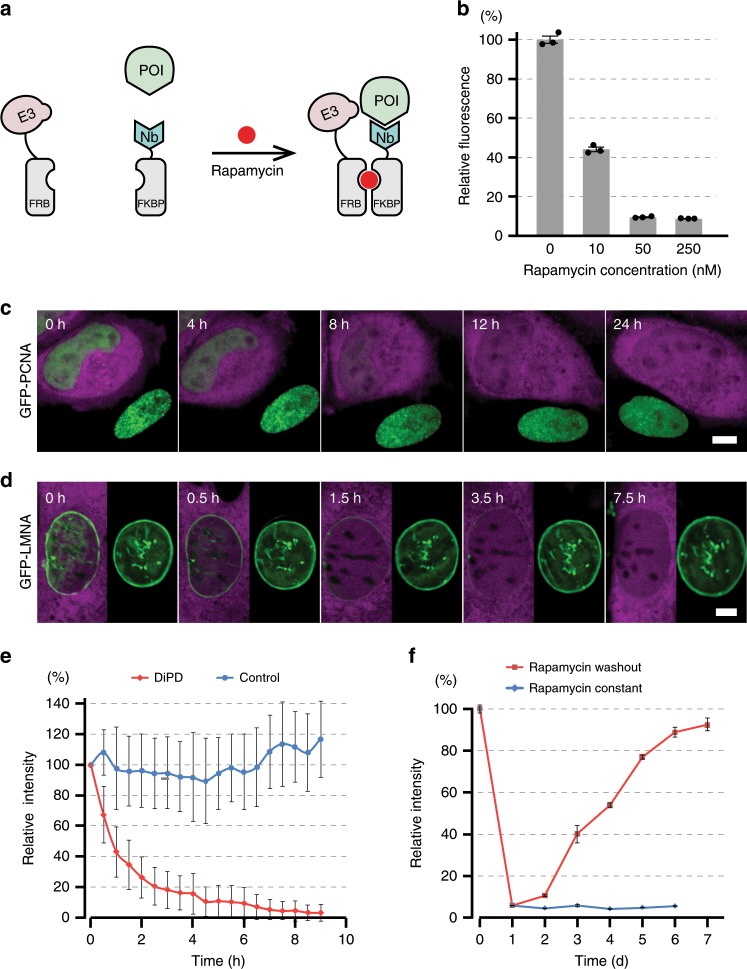
Drug-induced depletion of GFP fusion proteins. **a** Principle of drug-induced protein depletion. The destruction module (E3, ubiquitin E3 ligase) and the targeting module (Nb, Nanobody) are fused to FRB and FKBP, respectively. Upon addition of rapamycin FRB and FKBP heterodimerize triggering the ubiquitination and depletion of the POI (protein of interest). **b** Dose dependency of GFP-PCNA depletion. Biological triplicates were measured and results are shown as mean ± SD. **c**, **d** Snapshots of GFP-PCNA (**c**) and GFP-LMNA (**d**) depletion after rapamycin treatment. GFP fusion proteins are shown in green and the DiPD system is marked with DsRed (in magenta). Scale bars are 5 µm. **e** Kinetics of GFP-LMNA depletion after rapamycin treatment. Measured DiPD cells *n* = 15 and controls without DiPD *n* = 11, error bar stands for SD. **f** The recovery of protein levels after rapamycin removal. Biological triplicates were measured and results are shown as mean ± SD.

To obtain a second tool that can be used independently or in combination with this LiPD system, we developed a drug-induced protein depletion (DiPD) tool. We utilized a chemically induced dimerization (CID) pair to bring the targeting and destruction modules into close proximity. Similar to the LiPD, the POI is contacted by a specific targeting module that is fused with one half of the CID pair. Upon drug addition, this targeting module heterodimerizes with the destruction module via the complementary CID half causing the POI to be ubiquitinated and thus marked for proteasomal degradation (Fig. 2a). Three CID pairs, induced with abscisic acid (ABA)^23^, gibberellic acid (GA-3)^24^, or rapamycin^25^, were tested but only the rapamycin induced FKBP/FRB pair showed efficient depletion of the POI (Supplementary Fig. 6a). To optimize the efficiency of the FKBP/FRB-based DiPD system we tested different configurations and linker lengths. The direct comparison showed that the target (GFP-PCNA) was most efficiently depleted by the GBP5-FKBP connected via a 2× or 3× GGGS linker (Supplementary Fig. 6b, c). Analogous to the LiPD, the two optimized DiPD parts were placed in a piggyBac transposon vector for efficient transposase mediated genome integration (Supplementary Fig. 6d). To control for possible protein depletion caused by the single components, the E3 ligase or rapamycin, GFP-PCNA cells were transfected with the E3FRB catalytic construct (without the FKBP part) and then treated with rapamycin. The comparison shows that all three components for catalysis, targeting, and induction are required for efficient protein depletion (Supplementary Fig. 6e).To systematically test the performance of the DiPD, the DiPD piggyBac vector was integrated in a HeLa cell line stably expressing GFP-PCNA. Firstly, we examined whether the stably expressed DiPD system itself disturbs the abundance of cellular GFP-PCNA. The GFP-PCNA cells with DiPD were mixed with cells lacking the system, and imaged for high-throughput analysis. The comparison with control cells shows that in the absence of rapamycin induction the stable expression of DiPD does not affect GFP-PCNA levels (Supplementary Fig. 6f).

To test the dose dependency of protein depletion, we incubated the cells with different rapamycin concentrations for 12 h and then measured average GFP-PCNA intensities in bulk with a fluorescence plate reader. Already 10 nM rapamycin was sufficient to deplete the target protein below 50% and higher concentrations (50 and 250 nM) achieved a depletion to less than 10% of the original protein levels (Fig. 2b). As no adverse side-effects, such as cell death, cell cycle arrest, or abnormal morphological changes, were observed, we chose 250 nM for subsequent applications and the corresponding time course images showed no detectable target protein after 8 h (Fig. 2c).

Next, we tested the DiPD with a stable protein and chose nuclear LaminA (LMNA), which has a reported half-life of about 4 d in cultured cells^26^. In mouse embryonic fibroblast (MEF) cells, the stably expressed GFP-LMNA was rapidly degraded with a half depletion time of about 1 h and within 4 h more than 95% of the target protein was depleted while no loss was detected in cells without DiPD (Fig. 2d, e and Supplementary Movie 5).

To evaluate the sustainability and reversibility of DiPD, we tracked GFP fluorescence intensities in cells cultured in the presence of rapamycin or with rapamycin withdrawn after 24 h. The cellular GFP-PCNA could be maintained at the barely detectable level of below 10% in the presence of rapamycin and gradually recovered after removal of rapamycin, demonstrating both sustainable and reversible depletion of the POI (Fig. 2f).

Besides nuclear proteins, we also tested the DiPD system for depletion of cytosolic proteins like the Isocitrate dehydrogenase 1 (IDH1) which catalyzes the decarboxylation of isocitrate. We generated a HeLa cell line expressing GFP-IDH1 together with the DiPD system. Upon rapamycin induction, a rapid depletion of the cytosolic GFP-IDH1 was observed (Supplementary Fig. 7). Similarly, another cytoplasmic enzyme adenosylhomocysteinase (AHCY) could be depleted via the DiPD system within an even shorter time period, demonstrating efficient degradation of cytoplasmic proteins with the DiPD system. Likewise, we tested different type of transmembrane proteins for depletion with the DiPD system. We could show inducible depletion of the C-terminal GFP fusion of three-prime repair exonuclease 1 (TREX1) protein, which localizes on the endoplasmic reticulum (ER) membrane via a single transmembrane helix and of the inner nuclear membrane MAN1 protein, which utilizes a double transmembrane helixes. In addition, we demonstrated depletion of the Lamin B receptor (LBR) protein, which is anchored in the ER and nuclear membrane via eight transmembrane helixes (Supplementary Fig. 7). In comparison to nonmembrane proteins, depletion of integral transmembrane proteins was slower, which likely reflects their retention from proteasomal degradation. An accelerated degradation of these membrane proteins could be observed after cell division, suggesting that the accompanying reorganization of cellular membranes partially mobilizes transmembrane proteins and increases chances for their proteasomal degradation.

To expand the general applicability of DiPD, we designed a versatile DiPD vector for the simple insertion of any targeting modules. We validated this vector system with a GFP binding designed ankyrin repeat protein (DARPin, 3G124nc)^27^ and an mCherry-binding nanobody LaM4^28^ (Supplementary Fig. 8a) to target GFP- and mCherry- fusion proteins, respectively. Both, nanobody and DARPin based, DiPD constructs allowed for efficient and rapamycin dependent depletion of GFP- or mCherry- fusion proteins as demonstrated with GFP-PCNA (Supplementary Fig. 8b, c) or mCherry-LMNA (Supplementary Fig. 8d).

### Drug-induced depletion of endogenous proteins

In all protein depletion experiments thus far we targeted fluorescent fusion proteins, which allows direct monitoring of depletion efficiency and provides a quantitative, temporal and spatial correlation of target protein levels with cellular phenotypes at single cell resolution. However, the insertion of artificial sequences coding for fluorescent fusion proteins can affect gene expression and/or protein function and requires lengthy genetic engineering procedures including selection and passaging of cells, which are not suitable for the study of primary cells or organisms without genetic modifications.

To deplete endogenous proteins, we generated DiPD vectors with nanobodies binding to nuclear LaminA/C (LMNA/C) and PCNA protein (Supplementary Fig. 9a). Immunofluorescence showed that the endogenous LMNA/C protein was efficiently depleted from the nuclear envelope in cells containing the DiPD after rapamycin induction (Supplementary Fig. 9b). PCNA is an interesting target as it is a central and essential DNA replication factor and knockout cells are not viable. HeLa cells with and without the PCNA targeting DiPD system were mixed and jointly treated with rapamycin for side-by-side comparison. Immunostaining after overnight incubation (12 h) demonstrated that the endogenous PCNA was efficiently depleted to background levels in a DiPD and rapamycin dependent manner (Supplementary Fig. 9c, d).

**Fig. 3:**
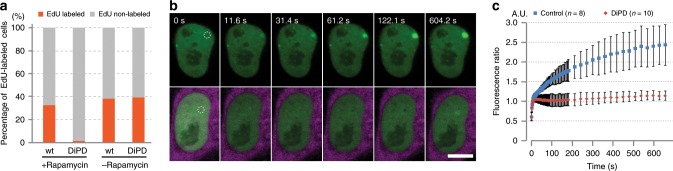
Transient depletion of an essential protein with DiPD. **a** EdU labeling of DNA synthesis in cells after endogenous PCNA depletion. Practically no EdU incorporation was detected in induced DiPD expressing cells, while around 1/3 of cells without DiPD (wt) and/or without rapamycin induction were EdU positive. For each group, around 1200 to 6000 cells were analyzed. **b** Recruitment of GFP-tagged DNA LIG1 to DNA damage repair sites in the presence and absence of PCNA. Recruitment of DNA LIG1 (green) to the DNA damage repair site (dashed circle) was abolished after PCNA depletion (lower panel, DiPD shown in magenta), but not in cells with PCNA (upper panel). Scale bar equals to 10 µm. **c** DNA LIG1 recruitment kinetics in cells with and without PCNA. Results are shown as mean ± SD.

This transient depletion of the essential protein PCNA now permits direct functional studies. As PCNA is the central loading platform for a number of factors at nuclear replication foci^29^, we monitored DNA synthesis with an EdU (5-ethynyl-2´-deoxyuridine) labeling assay. EdU is a nucleotide analog, which is readily incorporated into newly synthesized DNA at nuclear replication sites. However, upon rapamycin induced PCNA depletion no EdU incorporation could be detected (Fig. 3a and Supplementary Fig. 9e).

Besides its essential role in DNA replication, PCNA also participates in DNA damage repair (DDR) pathways building a dynamic loading platform for other factors like, DNA ligase 1 (LIG1), which ligates DNA strands during DNA replication and damage repair^30,31^. The role of PCNA in recruitment of cellular factors to nuclear repair sites has mostly been investigated by mutating the PCNA binding domains (PBDs) of these factors^31^. To directly study the role of the essential PCNA, we applied our DiPD system and measured the recruitment of LIG1. We inflicted DNA damage at defined nuclear spots by laser microirradiation and monitored the recruitment of GFP-LIG1 in cells with and without PCNA depletion. While in undepleted cells LIG1 is rapidly recruited to DDR sites within minutes, practically no accumulation was detected in cells upon rapamycin induced depletion of PCNA (Fig. 3b and Supplementary Movie 6). The quantification shows an accumulation of GFP-LIG1 at DDR sites starting a few seconds after microirradiation and increasing during the following minutes, while only minor GFP-LIG1 recruitment was detected upon depletion of the endogenous PCNA (Fig. 3c). These results demonstrate how the DiPD system enables the study of essential proteins as illustrated with these functional studies of PCNA in DNA replication and repair.

### Multiple and combinatorial depletion of proteins

**Fig. 4:**
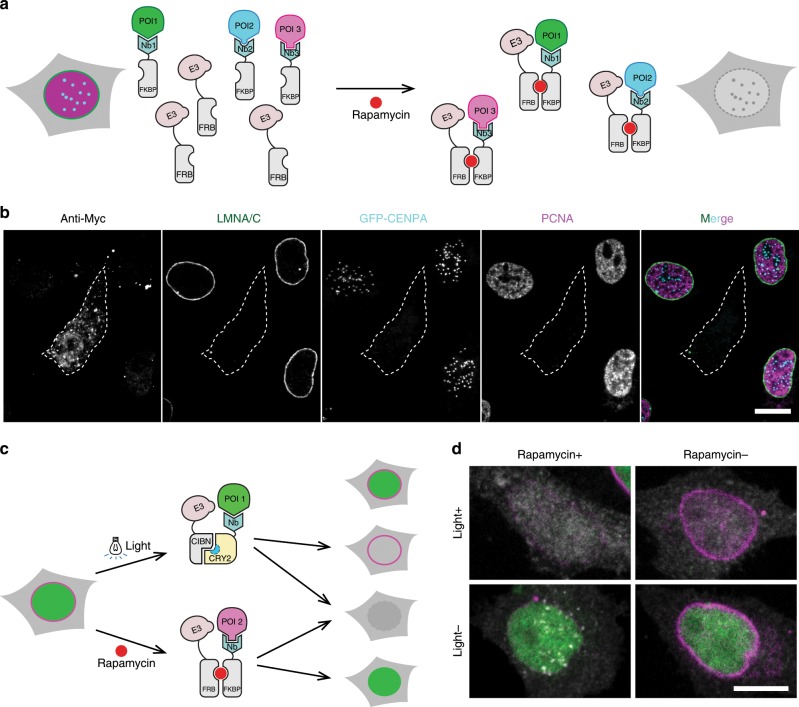
Combinatorial depletion of different sets of multiple proteins. **a** Schematic outline of the multi-protein targeting DiPD system. Multiple POIs (proteins of interest, POI1-3) are targeted by distinct protein specific targeting modules (Nanobodies, Nbs), which are embedded in the DiPD system. Upon induction with rapamycin the POIs are ubiquitinated and degraded. E3 ubiquitin E3 ligase. **b** Triple depletion of endogenous LMNA/C, PCNA and GFP-CENPA proteins. GFP-CENPA cells were transfected with DiPD constructs targeting LMNA/C, PCNA and GFP. Proteins were detected by immunostaining or GFP fluorescence after rapamycin treatment. A triple depletion of targeted proteins could be observed in the presence of rapamycin. **c** Principle of combinatorial light and drug-induced depletion of two independent proteins (POI1 and POI2). **d** Combinatorial depletion of a nucleoplasmic (GFP-CXXC4, in green) and a nuclear envelope protein (LMNA/C, in magenta). Cells with the light and drug inducible protein depletion tools express DsRed as marker (in gray). Scale bars are 10 µm. Extended versions of this figure including uninduced controls are shown in Supplementary FigS. 10 and 11.

As most cellular processes involve multiple redundant proteins or alternative pathways, we set out to expand the range of our protein depletion systems. Since the destruction module of the DiPD system is an independent component, it can be easily combined with multiple targeting domains. To test the feasibility of such a multi-DiPD system we combined three different targeting modules binding LMNA/C, PCNA, and GFP (Fig. 4a). While the abundance of these targeted proteins was not affected in the absence of rapamycin, all three POIs were depleted after rapamycin treatment (Fig. 4b and Supplementary Fig. 10a, b).

While the multi-DiPD tool allows simultaneous depletion of multiple proteins, some biological questions may require the selective and combinatorial or sequential depletion of distinct target proteins. Towards this goal we combined our DiPD and LiPD systems to selectively deplete different sets of proteins with rapamycin and/or light induction. With LiPD we targeted the stably expressed GFP-CXXC4 and with DiPD the endogenous LMNA/C protein (Fig. 4c). We selectively applied the chemical and physical inducers (rapamycin and/or light) and obtained specific and combinatorial depletion of GFP-CXXC4 and LMNA/C (Fig. 4d and Supplementary Fig. 11). These results show that the combination of LiPD and DiPD allow the selective, spatial, and temporal depletion of different sets of proteins.

### Protein depletion in *C. elegans*

Next we applied targeted protein depletion to study protein function in the context of an entire organism. Genetic and physical manipulation by itself can easily impair cellular function and even cause death. Therefore, we aimed with our DiPD for the opposite i.e., the rescue of cells from programmed cell death. Since the ubiquitin-proteasome system exists in all eukaryotes, we picked *Caenorhabditis elegans*, which is a well- established model for apoptosis^32,33^. As a target, we chose the caspase CED-3, which is essential for triggering apoptosis in defined cells during *C. elegans* development. In animals with a *ced-3* loss of function *(lf)* mutation, cells that normally undergo apoptosis inappropriately survive causing an extracells phenotype.

Importantly, for targeted protein depletion to work, the RING domain of the mammalian E3 ligase has to functionally interact with an endogenous E2 enzyme of the host. Sequence comparison showed that Ube2d2, one of the ubiquitin conjugating E2 enzymes for the mouse RING^Lnx1^, is ubiquitously expressed and highly conserved from *C. elegans* to human (Supplementary Fig. 12a). To adapt DiPD for *C. elegans* we optimized the codon usage of the targeting and destruction components and used the ubiquitous *eft-3* promoter together with the *gpd-2/gpd-3* intergenic region for proper stochiometric expression (Supplementary Fig. 12b).

**Fig. 5:**
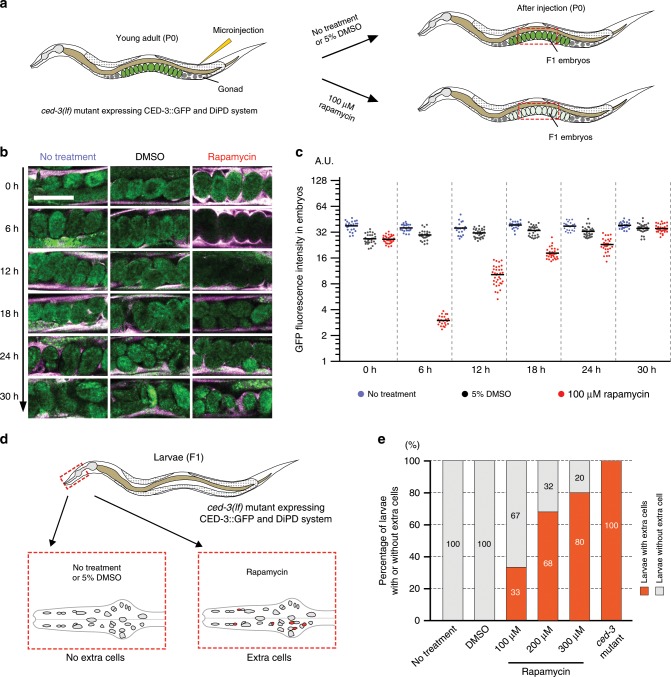
Transient depletion of CED-3::GFP transforms cell fate. **a** Schematic for drug-induced depletion of CED-3::GFP in *ced-3(lf)* embryos carrying the DiPD system. Rapamycin (100 µM) or DMSO (5%; control) was administered to adults (P0 generation) and CED-3::GFP fluorescence signal was monitored in embryos (F1 generation) over time. **b** Images of CED-3::GFP signal (in green) in embryos at different time points after rapamycin treatment. Scale bar 50 µm. **c** Quantification of CED-3::GFP signal in embryos. Data points and the mean values are shown. *n* = 19-34, biological replicates (embryos). **d** Schematic of rescue assay of extracells phenotype in the pharynx of *ced-3(lf)* mutants. **e** Rapamycin-mediated depletion of CED-3::GFP results in larvae (F1 generation) with extra cells. For each group 15–25 larvae were analyzed.

To be able to directly monitor the efficiency and biological effect of protein depletion in developing animals we generated transgenic animals where the endogenous CED-3 was functionally replaced by a GFP-tagged CED-3 (CED-3::GFP). The optimized GBP nanobody based DiPD system was then added as extrachromosomal array (*bcEx1328*). Rapamycin treatment (Fig. 5a) led to an almost complete depletion of CED-3::GFP in embryos within 6 h, which was not observed with the DMSO solvent control (Fig. 5b). The CED-3::GFP signal gradually recovered after 6–30 h (Fig. 5b, c), which might be due to inactivation of rapamycin in *C. elegans*.

To resolve protein depletion kinetics, we monitored CED-3::GFP levels in the first 6 h after induction (Supplementary Fig. 13a). Quantitative analyses showed a clear reduction of CED-3::GFP already at the first time point (1 h) and maximal depletion between 4 and 6 h after induction (Supplementary Fig. 13b). We also tested two higher concentrations of rapamycin and found a dose-dependent protein depletion efficiency (Supplementary Fig. 13c, d).

Next, we wanted to investigate whether the rapamycin-mediated depletion of CED-3::GFP protein affects apoptosis and causes an extracells phenotype. To that end, we allowed the CED-3::GFP embryos to develop into larvae and analyzed their anterior pharynxes where the ‘extra cells’ phenotype can easily be scored (Fig. 5d). We found that none of the larvae from untreated or DMSO-treated embryos exhibited an ‘extra cells’ phenotype. In contrast, we found that depending on the concentration of rapamycin, from 33 to 80% of larvae from rapamycin-treated embryos exhibited an extracells phenotype, indicating that in these animals, CED-3::GFP was depleted below the level required for apoptosis (Fig. 5e). These results demonstrate that DiPD is well suited to study protein function in an intact, developing organism and can even be used to achieve positive outcomes such as the rescue of cells from apoptotic death.

## Discussion

A comprehensive understanding of cellular processes requires an inventory of all cellular components and studies to identify their function in the context of cells and organisms. Genetic methods to inactivate or mutate target genes typically feature high precision and complete penetrance but tend to be rather static. In contrast, knockown methods targeting mRNA or protein levels are by nature less precise but far more dynamic. While siRNA and shRNA based methods have been enhanced for specificity and efficiency, they still depend on the natural turnover of the corresponding proteins, which may take days and weeks until any effect can be seen.

Here we present a versatile toolset for the direct depletion of cellular proteins. The goal was to combine the flexibility to target virtually any cellular structure with temporal and spatial control. For many applications it is desirable to work with fluorescently tagged proteins as long as the genetic manipulation does not affect expression or function. The use of fluorescent tags permits monitoring of cellular protein levels and their subcellular localization over cell division cycles and in response to external stimuli. Therefore, we started with existing cell lines expressing fluorescent fusions to directly monitor and optimize our protein depletion strategies (DiPD and LiPD), which has the additional advantage that the validated targeting constructs described here will cover a large share of future applications and alleviate the need to develop new binders or constructs.

In many cases, it is, however, necessary or desirable to directly target the endogenous proteins without the need for prior genetic engineering and lengthy selection procedures, which are often not possible or perturbing in studies of primary cells. Here we demonstrate that our inducible protein depletion strategy can be applied to endogenous proteins and that different binders like nanobodies and DARPins may be used for targeting. Until recently, the intracellular application of recombinant binders has been hampered by their limited availability but the development of new screening technologies and the establishment of complex synthetic libraries now allow for rapid identification of new binders including nanobodies, monobodies, affimers, and DARPins^28,34–40^. Over the past years, there has been a steep increase in binders against cellular target structures including post-translational modifications as well as alternative protein conformations and activity states^37,41–43^. In addition, naturally occurring protein-binding domains could be repurposed. The modular design of our vectors accommodates and facilitates the use of all kinds of binding domains to target in principle any cellular structure.

The transient nature of this targeted protein depletion enables studies of essential proteins as we demonstrated with PCNA, which is strictly required for DNA replication in S phase. With rapamycin induced depletion of the endogenous PCNA we opened a window for functional studies and could analyze the role of PCNA in DNA repair. The ability to gradually tune protein levels with DiPD and LiPD also provides quantitative data for model refinements, to identify rate-limiting steps and protein levels and to explore genetic phenomena like haploinsufficiency.

As the ubiquitin-proteasome system exists in all eukaryotes, archaea and also in some bacteria (*Actinomycetales* and *Nitrospirales*)^44^, our toolset for targeted protein depletion should be broadly applicable. As one example, we successfully used the DiPD to test the role of CED-3 in the control of apoptosis in *C. elegans* and could show that transient depletion of CED-3 rescues daughter cells from programmed cell death. For chemical induction we used rapamycin as it binds with high affinity and efficiently induced protein depletion. In our cellular systems we did not observe any adverse side effects and used even higher concentrations up to 500 nM. To reduce or avoid mTOR inhibition also rapamycin derivatives (rapalogs) could be used^45^ to control dimerization and targeted protein depletion.

Our protein depletion toolsets use light and rapamycin as inducer and can therefore be combined with auxin driven AID or PROTAC systems allowing for independent control of three or more sets of proteins. Also, in our first tests the plant hormones ABA and GA-3 were far less efficient inducers for targeted protein depletion. However, these small molecules had been successfully used to induce heterodimerization in mammalian cells^23,24,46^, suggesting that the respective fusion constructs we had used for DiPD might be less active due to steric constrains. Therefore, with a systematic optimization of linker lengths, fusion points and domain sizes also these plant hormones should function in DiPD. Altogether, our light and rapamycin induced protein depletion toolsets in combination with auxin, ABA and GA-3 driven depletion tools allow independent control of multiple sets of proteins and should help to elucidate the interplay or co-dependence of proteins and cellular pathways.

The modular design of these toolsets also allows for an easy exchange of the catalytic domain to switch from ubiquitin ligation to other post-translational modifications like e.g., targeted phosphorylation, acetylation or methylation to name only a few. In other words, these toolsets for protein depletion could easily be expanded to control practically any post-translational modification and its removal. To avoid any genetic alterations, the protein depletion or modification tools could also be introduced as mRNA or directly as proteins using either cell penetrating peptides, mesoporous beads or other protein delivery methods^47,48^. In this study we target proteins for basic research, but viral or pathogenic proteins or oncoproteins could also be targeted for therapeutic purposes.

In summary, we present versatile toolsets for light and drug-controlled targeted protein depletion, which enables the independent temporal and spatial control of two or more sets of proteins in cells and organisms.

## Methods

### Cell culture

HeLa (WT and transgenic) cells and BHK cells^49^ were cultured in DMEM (Sigma) supplemented with 10% FBS (Fetal Bovine Serum, Sigma), mouse embryonic fibroblast cells (MEFs) were cultured in DMEM with 15% FBS. All cells were cultured under 5% CO_2_ at 37 °C. For live cell imaging, cells were cultured in phenol-red free DMEM supplemented with FBS. The microscope stage was warmed up to 37 °C and supplied with humidified air containing 5% CO_2_.

### Plasmid construction

Gene fragments, encoding FBW1A, FBW1B, KEAP1, and LNX1 functional domains, were cloned from cDNA derived from mouse J1 embryonic stem cells. The HECT domain of NEDD4 was amplified from pCI-neo.mCherry-NEDD4^50^. The E3 ligase fragments together with GBPs^41^ were cloned into pIRES2-DsRed Express plasmid (Clontech). For fluorescent fusion proteins, coding sequences for LaminA, Man1, Lbr, Ahcy, and IDH1, were amplified from mouse cDNA, and fused to the C-terminus of eGFP or mCherry. TREX1-GFP and GFP-CXXC4 constructs were published before^51,52^. Sequences encoding mCherry-binding nanobody LaM4 and GFP binding DARPin 3G124nc were synthesized (Eurofins Genomics) according to published data^27,28^.

The plasmid pBC1661 (P_*ced-3*_*ced-3::gfp*) used to express CED-3::GFP in vivo was generated by dividing the full length of *ced-3* rescuing fragment into two fragments (~6 kb + 7 kb) and amplifying both fragments from genomic DNA using PCR. The two fragments were ligated into the pCFJ909 (miniMos vector) using the Gibson assembly kit (New England Biolabs). To construct the pDiPD-GBP5_worm (P_*eft-3*_*ring::frb::ha::gpd-2/gpd-3::gbp5::fkbp::myc*_3’UTR) plasmid, codon optimized gene fragments encoding RING^Lnx1^, GBP5, FKBP and FRB with artificial introns were synthesized (Eurofins Genomics) and cloned using the Gibson assembly kit (New England Biolabs).

All primers used in this study are listed in Supplementary Table 1; all plasmids used are listed in Supplementary Table 2.

### Cell lines and worm strains

HeLa GFP-PCNA cells were from Chagin et al.^53^, MEF cells expressing GFP-LaminA were from Chiu et al^54^. To establish HeLa stably expressing GFP fusion proteins, cells were transfected with pCAG-GFP-CXXC4-IB plasmids with Lipofectamine 3000 (Thermo Fisher Scientific) following manufacturer’s instruction, selected with blasticidin S (Sigma–Aldrich) at the concentration of 6 µg/ml for 7 days, and purified with FACS (FACSAria II, BD Biosciences). MEF cells expressing mCh-LaminA were established by random integration after transfection of pPGK-mCh-LaminA plasmid, positive cells were purified by FACS.

For generating a GFP-CENPA knock-in cell line, a MIN-tagged CENPA HeLa cell line was firstly generated by insertion of an *attp* recombination site (MIN-tag)^22^ after the start codon of *CENPA* gene. Plasmids encoding pSpCas9A (pSpCas9(BB)-2A-GFP, addgene #48138)^55^ and an sgRNA targeting to *CENPA* start codon (5’-GCACCCTCTGCGGCGTGTCA-3’) together with a synthesized 200 nt ssDNA repair template, which contains homology arms centered around the MIN-tag were transfected into HeLa cells. Transfected cells (marked by GFP expression) were enriched by FACS (Aria II, BD Biosciences), and seeded into a P100 plate. After 8 days, single colonies were manually picked and transferred into a 96-well plate and expanded. Genomic DNA from each colony was isolated and screened by PCR using the MIN-external primers (primer sequences in Supplementary Table 1). To recombine GFP-CENPA into the genome, the MIN-CENPA cell line was transfected with the attb-GFP-CENPA plasmid together with the BXB1 integrase plasmid^56^. GFP-positive cells were selected 24 h after transfection with G418 (1 mg/ml, Sigma–Aldrich) and sorted by FACS. Enriched GFP-positive cells were seeded into P100 dish and expanded. GFP-positive colonies were picked manually under a fluorescence microscope, then expanded and characterized by screening PCR using the primers shown in Supplementary Fig. 4 and Table 1. The GFP-CENPA HeLa Kyoto cell line was further verified by Sanger sequencing of the recombined locus.

To generate DiPD and LiPD stable lines, the piggyBac-DiPD or piggyBac-LiPD plasmid was co-transfected with piggyBac transposase expression plasmid in a ratio of 3:1, and cells were selected with 150 µg/ml hygromycin (Sigma–Aldrich) for 7 days followed by FACS. Expression of the DiPD and LiPD units were confirmed by immunostaining with antibodies against the HA (3F10, Sigma–Aldrich, 1:200) and Myc-tag (9E10, Thermo Fisher Scientific, 1:200).

*C.elegans* strains were maintained at 20 °C. The following *C.elegans* strains were used and maintained at 20 °C: LGIV *ced-3(n717)*^57^; LGV *bcSi36* and *bcEx1328* (this study, Supplementary Table 3).

Germline transformation was performed as described^58^. For the miniMos injection, plasmid PBC1611 was injected at a concentration of 10 ng/µl together with the co-injection markers pCFJ601 at 50 ng/µl, pGH8 at 10 ng/µl, pCFJ90 at 2.5 ng/µl, pCFJ104 at 5 ng/µl into the miniMos strain HT1593 as described before^55^. A single copy of the *ced-3::gfp* transgene was integrated on chromosome V (LGV *bcSi36*) and the site of insertion was mapped and sequenced using inverse PCR^59^. For the extra-chromosome array injection, plasmid pDiPD-GBP5_worm (P_*eft-3*_*ring::frb::ha::gpd-2/gpd-3::gbp5::fkbp::myc*_3’UTR) was injected at a concentration of 50 ng/µl together with the co-injection markers P_*eft-3*_*mkate2* and pRF4 at 150 ng/µl into LGV *bcSi36*(P_*ced-3*_*ced-3::gfp*) strain to generate a stable extrachromosomal array *bcEx1328*.

All cell lines used are listed in Supplementary Table 3.

### Fixed sample slides preparation for confocal microscopy

To prepare sample slides for confocal microscope, cells were seeded on coverslips in 6-well plates. After transfection, induction or other treatments, cells were fixed with 4% formaldehyde in PBS (phosphate buffer solution) for 10 min at room temperature (RT), then washed three-times with PBST (PBS with 0.02% Tween). To permeablize the cell, fixed samples were treated with PBS containing 0.5% Triton X-100 for 5 min at RT, followed by three-times washing with PBST.

For samples with immunostaining, primary antibodies were diluted in blocking buffer (3% BSA in PBST), and incubated with the sample for 1 h at RT. Samples were washed three times with PBST, and incubated with fluorescein labeled secondary antibodies that are diluted in blocking buffer. One hour later, cells were washed three times with PBST to remove the unbound secondary antibodies.

Cell nuclei were stained with DAPI (4’,6-diamidino-2-phenylindole, 200 ng/ml in PBS) for 10 min at RT. Coverslips were mounted with Vectashield antifade mounting medium (Vector Laboratories) and sealed with clear fingernail polish on glass slides.

### Testing the depletion efficiency of E3 ligases

HeLa cells stably expressing GFP-CXXC4 or GFP-PCNA were seeded on 18 × 18 mm^2^ coverslips and transfected with pFBW1A-GBP1-IR, pFBW1B-GBP1-IR, pKEAP1-GBP1-IR, pLNX1-GBP1-IR, pGBP1-NEDD4-IR, and pGBP1-IR with Lipofectamine 3000 (Thermo Fisher Scientific). Cells were fixed and sample slides prepared. Image acquisition was performed with a spinning disc confocal microscope (Ultraview VoX, Perkin Elmer) using a ×63 objective and an EMCCD camera (1000 × 1000 pixels, Hamamatsu). For the analysis, cells were segmented according to the DAPI stained nuclei, and the intensities of GFP and DsRed signals in the nuclear segment of each cell were determined and average gray values calculated. The background signal was subtracted and the average GFP intensity of transfected cells (~20 cells) for each construct was calculated and plotted with RStudio program. pGBP1-IR transfected cells served as negative control.

### Association and dissociation of different LID pairs

BHK cells were seeded in 8-well µslides (ibidi GmbH) and transfected with plasmids encoding fluorescent proteins fused to the LID components SspB/iLID^20^ or PHR/CIBN^21^. A spinning disc confocal microscope (Ultraview VoX, Perkin Elmer) was used for live cell imaging and a 488 nm laser (600 ms, 10% of a 2.5 mW power) was used for heterodimers induction. Association and dissociation of the mCherry tagged LID components to and from the cell membrane anchored components was imaged with a 561 nm laser.

To check the association and dissociation of the PHR/CIBN heterodimer in the nucleus, BHK cells were seeded in 8-well µslides (ibidi GmbH), and triple transfected with pNLSGFP-iRFP670-PCNA, pCIBN-GBP and pCRY2PHR-mCherry plasmids. Fluorescence excited with 488 nm (for eGFP illumination and induction), 561 nm and 640 nm lasers was recorded at maximum imaging speed of the microscope. For the dissociation assay, only 561 nm laser and 640 nm lasers were used, and images were recorded using a preset program (maximum speed for 1 min, then 5 s intervals for 2 min, then 10 s intervals for 2 min, and 30 s intervals for 10 min). For data analysis, PCNA foci were segmented according to iRFP670 intensity ($I_{{\mathrm{PCNA}}}^{{\mathrm{iRFP}}670}$) and the PHR-mCh intensity at these foci was measured and average mCherry intensity values were calculated for each cell ($I_{{\mathrm{PCNA}}}^{{\mathrm{mCh}}}$). For each time point, the PHR-mCh associated with PCNA foci was calculated with Eq. (1).

1 $$I = \frac{{I_{{\mathrm{PCNA}}}^{{\mathrm{mCh}}} - I_{{\mathrm{NUC}}}^{{\mathrm{mCh}}}}}{{I_{{\mathrm{PCNA}}}^{{\mathrm{iRFP}}670} - I_{{\mathrm{NUC}}}^{{\mathrm{iRFP}}670}}}$$

$I_{{\mathrm{NUC}}}^{{\mathrm{mCh}}}$ and $I_{{\mathrm{NUC}}}^{{\mathrm{iRFP}}670}$ was the nuclear average intensity of mCherry and iRFP670, respectively.

### Optimization of the LiPD system

To test the different combinations for LiPD, HeLa cells stably expressing GFP-PCNA were seeded in 8-well µslides (ibidi GmbH) and co-transfected with three combinations for LiPD (GBP1-PHR/CIBN-RING, CIBN-GBP1/RING-PHR, GBP1-PHR/RING-CIBN). To test the efficiency of different GBPs for the LiPD, LiPD constructs using GBP1, GBP2, and GBP4 were transfected into GFP-PCNA cells. Cells were then imaged with a spinning disc confocal microscope (Ultraview VoX, Perkin Elmer). Four hundred and eighty-eight nanometer laser (10% of 2.5 mW for 0.6 s) was used for light induction and imaging GFP simultaneously. Five hundred and sixty-one nanometer laser was used to excite DsRed for identifying transfected cells. Image stacks were taken every 10 min (six frames per hour).

For data analysis, image stacks were projected with maximum intensity, and the GFP-PCNA intensity in the nuclear area was measured at time 0 min and 120 min for each group. Average intensity and the standard deviation were calculated and histograms were made in Excel (Microsoft).

### Testing the conditions for DiPD

HeLa GFP-PCNA cells were seeded on coverslips in 6-well plates and transected with DiPD plasmid or E3FRB-GBP5 plasmid. About 24 h after transfection, the cells were treated with 250 nM rapamycin or DMSO only overnight. Cells were then fixed and HA was detected by immunostaining with anti-HA antibody (3F10, 1:200, Sigma–Aldrich) and sample slides were prepared.

Sample slides were imaged with a spinning disc confocal microscope (Eclipse Ti, Nikon). GFP and Alexa Fluor 594 were excited with a 488 nm and 594 nm laser, respectively. 16-bits digital images were recorded with an EMCCD camera. Images were processed and quantified with imageJ. Measured fluorescence intensities for each group were plotted in boxplot format with R (Rstudio).

### Test of background depletion for LiPD and DiPD

For LiPD background depletion control, GFP-CXXC4 cells stably expressing LiPD were mixed with cells lacking the LiPD and seeded on coverslips. For testing of DiPD background control, GFP-PCNA cells with and without stably expressed DiPD were mixed and seeded on coverslips. Cells were fixed and HA was stained, microscopic slides were prepared as described before.

For high-content analysis, samples were imaged with an automatic fluorescence microscope (Operetta, Perkin Elmer). DAPI, GFP and Alexa Fluor 594 (anti-HA) were excited with correspondent lasers using a ×40 high NA objective. For each coverslip 121 fields were imaged (an 11 × 11 fields square area). Image analysis was performed with the Harmony analysis software (Perkin Elmer). In brief, cell nuclei were recognized and segmented in the DAPI channel and correspondent GFP and anti-HA fluorescence intensities were measured for each cell nucleus. The cells then were divided into two groups (LiPD or DiPD group versus control group) according to the anti-HA fluorescence intensity. The GFP intensity of each group was plotted in boxplot format with R (Rstudio).

### Depletion of transmembrane and cytoplasmic proteins

HeLa cells were transfected with the DiPD plasmid together with a plasmid encoding a PiggyBac transposase (System Biosciences) using Lipofectamine 3000 (Thermo Fisher Scientific). Cells stably expressing DiPD were selected with hygromycin at 150 µg/ml followed by purification with FACS (FACSAria II, BD Biosciences). To deplete the GFP fused membrane or cytoplasmic proteins, the stable DiPD cells were transiently transfected with a plasmid encoding the GFP fusion. Cells were cultured and selected with antibiotics (blasticidin S: 6 µg/ml; G418: 150 µg/ml), then cell mixtures were seeded into 8-well or 2-well µslides (ibidi GmbH). Cells were treated with 250 nM rapamycin and imaged immediately with a spinning disc confocal microscope (Eclipse Ti, Nikon) equipped for live cell culture (with heating and humidified CO_2_ supply). GFP and DsRed were excited with 488 nm and 561 nm lasers, respectively, and cells were imaged every 30 min for about 24 h. Acquired images were processed and organized with imageJ.

### Detection of endogenous PCNA depletion by immunostaining

HeLa cells expressing the DiPD-aPCNA-IR (with DsRed marker) were mixed with wt HeLa cell at a 1:1 ratio and seeded onto coverslips in a 6-well plate. Cells were cultured in medium containing 250 nM rapamycin for about 12 h, and then fixed with 4% formaldehyde in PBS for 10 min. Cells were permeabilized in PBS with 0.5% TritonX-100 for 5 min and blocked in the blocking solution (PBS with 3% BSA, 0.2% Tween) for 1 h at room temperature. Rat anti-PCNA (16D10, 1:10) supernatant and mouse anti-myc (1:200, 9E10, Thermo Fisher Scientific, MA1-980) were diluted in the blocking solution and incubated with the samples for 1 h at RT. Alexa Fluor 488 labeled donkey anti-rat (Life Technology, A21208) and Alexa Fluor 647 labeled donkey anti-mouse (Life Technology, A31571) secondary antibodies were diluted in the blocking solution at a ratio of 1:200. Secondary antibody incubation was performed at RT for 1 h. Cell nuclei were stained with DAPI (200 ng/ml in PBS), and the coverslips mounted in Vectashield antifade medium (Vector Laboratories) and sealed with clear nail polish.

### EdU labeling of DNA synthesis in HeLa cells

HeLa cells expressing the DiPD-aPCNA-IR were mixed with wt HeLa cell at a 1:1 ratio and seeded onto coverslips in 6-well plates. Then the cells were treated with 250 nM rapamycin for 24 h and incubated in pre-warmed medium containing 10 µM EdU (5-ethynyl-2′-deoxyuridine) for 15 min, then gently washed twice with PBS and fixed with PBS containing 4% formaldehyde. The fixed cells were permeabilized with 0.5% TritonX-100 in PBS and blocked with 3% BSA in PBST (PBS with 0.2% Tween). Click chemistry on the incorporated EdU was performed in the reaction cocktail (4 mM CuSO4, 50 nM sodium ascorbate and Alexa Fluor 488 labeled azide dye in 0.1 M Tris/HCl pH 7) for 1 h in a humidified dark chamber, then washed three times with PBST (PBS with 0.2% Tween). HA-tagged DiPD components were detected with rat anti-HA (3F10, 1:200, Sigma–Aldrich) monoclonal antibodies and the goat anti-rat Alexa Fluor 594 secondary antibody (1:200, Life Technology, A11007). The samples were incubated with the diluted antibodies for 1 h at RT in a humidified chamber. DNA was stained with 200 ng/ml DAPI in PBS. Coverslips were mounted in Vectashield antifade mounting medium (Vector Laboratories) and sealed with clear nail polish.

### Quantitative high-throughput assay of EdU labeling

Slides with mounted coverslips were imaged with the Operetta high-content imaging system (Perkin Elmer). DAPI, Alexa Fluor 488 dye and Alexa Fluor 594 dye were excited with correspondent lasers using a ×40 high NA objective. For each coverslip, 121 fields were imaged (an 11 × 11 fields square area). Image analysis was performed with the Harmony analysis software (Perkin Elmer). Cell nuclei were segmented according to the DAPI staining and then the average signal intensities obtained with 488 nm (EdU staining) and 594 nm (HA staining) excitation was quantified in these segmented areas of all cells. Based on their Alexa Fluor 594 signal, cells treated with or without rapamycin were divided into DiPD and control (wt) groups. Replicating cells in each group were identified by EdU-labeling.

### Light induced depletion of GFP-PCNA and GFP-CXXC4

HeLa cells stably expressing GFP-PCNA were seeded in 8-well µslides (ibidi GmbH) and transfected with the pVitro-LiPD-GBP1-IR plasmid. HeLa cells stably expressing GFP-CXXC4 and the LiPD were mixed with cells without LiPD system, and seeded in 8-well µslides (ibidi GmbH). Cell culture medium was changed to phenol-red free medium and balanced for CO_2_ in the incubator before imaging. The 488 nm laser was used to excite the GFP (600 ms, 10% of a 2.5 mW power) and induce the protein depletion simultaneously, 561 nm laser was used to detect the co-expressed DsRed signal. The induction and imaging frequency was 10 frames per hour (6 min interval between two frames), and cells were imaged for about 5 h. Z- stacks of the image were acquired to avoid a loss of focus during the long-term imaging process.

To quantify the GFP signal, z-stack projection with maximum intensity was performed and the average intensity of the cell nucleus was determined after background subtraction. To exclude potential GFP fluorescence loss due to photobleaching, all ratios were compared to non-transfected control cells in the same imaging field.

### Drug-induced depletion of GFP-PCNA and GFP-LaminA

Cells stably expressing GFP fusion proteins together with the DiPD were mixed with cells only expressing GFP fusion proteins (serving as control) and seeded in 2-well µslides (ibidi GmbH). Attached cells were imaged after addition of the inducer (250 nM rapamycin) with a spinning disc confocal microscope (Eclipse Ti, Nikon) equipped for live cell culture (with heating and humidified CO_2_ supply). GFP and DsRed were excited with 488 nm and 561 nm lasers, respectively, and cells were imaged every 20 or 30 min.

For image analysis, cell nuclei (for GFP-PCNA quantification) or nuclear envelope (for GFP-LaminA quantification) were segmented manually and the fluorescence intensities measured with ImageJ. The average gray values in these segmented areas of cells at each time point were plotted in Excel (Microsoft).

### Sustained or reversible depletion of GFP-PCNA

HeLa cells stably expressing both GFP-PCNA and the DiPD-GBP5-IR were cultured in 60 mm culture dishes. Cells were treated with 250 nM rapamycin for 24 h, then split into two wells, maintained in medium with rapamycin (rapamycin constant, for sustained DiPD) or without rapamycin (rapamycin washout, for reversible DiPD) for 7 d. Every 24 h, cells were trypsinized and collected for measurement of the GFP-PCNA fluorescence intensity. To measure the GFP and DsRed fluorescence, cells were trypsinized and spun down at 180 g for 5 min, washed twice with PBS, resuspended in 100 µl PBS and transferred to 96-well microplates (µCLEAR, black, Greiner Bio-one) for measuring the fluorescence intensity with a fluorescence plate reader (Infinite M1000, Tecan). For data analysis, fluorescence intensities of GFP-PCNA were normalized by the intensity of DsRed to adjust the cell number differences, then the GFP-PCNA intensity at each time points was plotted in Excel (Microsoft). Biological triplicates were performed.

### Laser induced DNA damage and LIG1 recruitment assay

HeLa cells expressing the DiPD-aPCNA-IR (with DsRed marker) were mixed with wt HeLa cell at a 1:1 ratio, and seeded in 2-well µslides (ibidi GmbH). Cell mixtures were transfected with GFP-LIG1 expression plasmid. Cells were incubated with 250 nM Rapamycin for 12 h to deplete cellular PCNA. Before laser microirradiation, the culture medium was changed to phenol-red free medium and cell mixtures were incubated with 10 µg/ml Hoechst 33342 for 10 min to sensitize the cell for DNA damage induction. Cells were imaged with an Ultraview Vox spinning disc confocal microscope, and DNA damage was induced with a 405 nm laser (spot irradiation with 50% laser intensity). GFP-LIG1 (excitated by 488 nm laser) and DsRed (excitated by 561 nm laser) were imaged at maximum speed with 2 s intervals between frames for 2 min, then 5 s intervals for 1 min followed by 30 s intervals for 10 min. Images were recorded with a frame size of 1000 × 1000 pixels and 109 nm per pixel.

For quantification of local enrichment at DNA damage sites, the mean intensity of GFP-LIG1 at the irradiated region (ROI) was measured as *I*_ROI_, and compared to the GFP-LIG1 intensity of the whole nucleus *I*_nuc_. The enrichment of GFP-LIG1 at the ROI equals *I*_ROI_- *I*_nuc_. To compensate for potential photobleaching during image acquisition, the reduction of whole nuclear GFP-LIG1 intensity at time point *T*n (*I*_nuc *T*n_) was compared to the intensity at the start point *T*_0_ (*I*_nuc *T*0_). The accumulation of GFP-LIG1 at the DNA damage site at time point *T*n (*I*_*T*n_) was normalized and calculated with Eq. (2).

2 $$I_{T{\mathrm{n}}} = \frac{{I_{{\mathrm{ROI}}\,T{\mathrm{n}}} - I_{{\mathrm{nuc}}\,T{\mathrm{n}}}}}{{I_{{\mathrm{ROI}}\,T0} - I_{{\mathrm{nuc}}\,T0}}} \ast \frac{{I_{{\mathrm{nuc}}\,T0}}}{{I_{{\mathrm{nuc}}\,T{\mathrm{n}}}}}$$

### Dose-dependent depletion of GFP-CXXC4 with a lightbox

For light induced depletion of GFP-CXXC4 across large cell populations, HeLa cells stably expressing GFP-CXXC4 and LiPD-GBP1-IR were seeded in p35 dishes and induced with an LED lightbox using four different induction programs (program 1-4) with alternating light and dark phases of different duration (program 1: t_1_ = 1 s, t_2_ = 5 min; program 2: t_1_ = 1 s, t_2_ = 2 min; program 3: t_1_ = 4 s, t_2_ = 2 min; program 4: t_1_ = 8 s, t_2_ = 2 min. t_1_ is the duration of light induction, and t_2_ is the interval in the dark between inductions, as shown in Supplementary Fig. 5f). For each program, at time points 0, 0.5, 1, 2, and 4 h after induction, cells were trypsinized, washed and collected, then GFP and DsRed fluorescence was measured with a fluorescence plate reader (Infinite M1000, Tecan), and plotted in Excel. The constitutive DsRed signals were used to normalize for cell numbers. Biological triplicates were performed for each program and each time point.

### Construction of the lightbox

A 470 nm LED light bulb (3 W, 30 lm, 10°, Ledxon) and a digital timer (Tempatron UDT, Eltime Controls) were used to build a lightbox, which could illuminate the cells programmably. The distance from the light bulb to cultured cells (30 cm) was chosen to ensure even sample illumination.

### Spatially controlled LiPD

Cells stably expressing GFP-CXXC4 and the LiPD system or without the LiPD (control) were seeded in 4-well µslides (ibidi GmbH) and cultured till full confluency. The cells were illuminated and imaged with a spinning disc confocal microscope (Eclipse Ti, Nikon) using a ×60 oil objective. Imaging fields arranged in a chess board pattern were illuminated with a 488 nm laser (300 ms, 0.7 mW per cm^2^) to achieve a spatial controlled depletion of GFP-CXXC4 in cells. Cells were induced repeatedly every 6 min for 4 h. After the four hours induction, the final fluorescence overview image was collected as a 7 × 7 large image field. The GFP-CXXC4 cells without the LiPD system were induced and imaged with the identical experimental setup to control for loss of fluorescence due to photobleaching.

### Multi-protein targeting DiPD

HeLa GFP-CENPA cells were seeded on coverslips in 6-well plates and triple transfected with DiPD-aLamin, DiPD-aPCNA, and DiPD-GBP plasmids (3 µg plasmid DNA per well, at the ratio of 1:1:1). Cells were cultured overnight and treated with rapamycin (250 nM) for 24 h and fixed with formaldehyde. Immunostaining was performed to detected the LaminA/C (with rabbit anti-LMNA/C antibodies, Millipore, 1:500), PCNA (with monoclonal rat anti-PCNA antibody, 16D10, supernatant, 1:10) and Myc- tagged nanobodies (with antibody mouse anti-Myc, 9E10, 1:200, Thermo Fisher Scientific). Alexa Fluor 405 labeled goat anti- mouse (A31553), Alexa Fluor 594 labeled goat anti- rabbit (A11034) and Alexa Fluor 647 labeled goat anti- rat (A21247) were used as secondary antibodies (all from Life Technology, 1:200). Coverslips were mounted on slides and fluorescence images were acquired with the spinning disc confocal microscope (Ultraview VoX, Perkin Elmer) as described before.

### Combinatorial depletion of multiple proteins

HeLa GFP-CXXC4 cells were cultured on coverslips in 35 mm culture dishes and co-transfected with LiPD-GBP1 and DiPD-aLamin constructs. 24 h after transfection, rapamycin was added into the culture medium to a concentration of 250 nM overnight (for rapamycin treatment group). Next, cells were put into the lightbox and induced with the 470 nm LED light for 4 s repeatedly every 2 min for light induced depletion of GFP-CXXC4. After 4 h of induction, cells were fixed and permeabilized, then stained with antibody against LaminA/C (rabbit anti-LMNA/C antibodies, Millipore, 1:500) and HA tag (3F10, 1:200, Sigma–Aldrich) to detect the LiPD or DiPD components. Cell nuclei were stained with DAPI and the coverslips were mounted on slides and sealed with clear nail polish. Cells were imaged with a spinning disc confocal microscope (Ultraview VoX, Perkin Elmer) as described above.

### Phenotype analysis and microscopy for worm experiments

Rapamycin and DMSO microinjections were performed as described^60^. Once adults were injected with different reagents (5% DMSO and 100 µM, 200 µM, 300 µM rapamycin or without injection), they were imaged using a Leica TCS SP5 II confocal microscope at 0, 1, 2, 3, 4, 5, 6, 12, 18, 24, and 30 h after injection. The slide preparation and all settings of the confocal to record GFP fluorescence signal were performed as described^61^. Briefly, a Z-stack volume of ~20–25 µm with a step size of 2.0 µm was setup to record the GFP fluorescent signal of the embryos in each injected adult (laser power was 15% for 488 nm and 1% for 561 nm). For all the confocal images, a noise reduction function was applied by using the Leica Application Suite (LAS) software to remove the cytoplasmic noise background.

Following confocal acquisition of CED-3::GFP fluorescence signal in the embryos of injected adults, for every Z-slice in which a distinct whole embryo boundary could be seen, the mean intensity of the CED-3::GFP fluorescent within the whole embryo boundary was determined by drawing the region of interest (ROI) around the boundary of the whole embryo in ImageJ.

To observe extra cells in anterior pharynx, injected adults (P0 generation) were maintained at 20 °C to recover treated F1 embryos. Once F1 embryos had developed into L4 stage larvae, the number of extra cells in the anterior pharynx was determined using Nomarski Optic as described^62^.

### Reporting summary

Further information on research design is available in the Nature Research Reporting Summary linked to this article.

## Supplementary information

Supplementary Information

Description of Additional Supplementary Files

Supplementary Movie 1

Supplementary Movie 2

Supplementary Movie 3

Supplementary Movie 4

Supplementary Movie 5

Supplementary Movie 6

Reporting Summary

## Data Availability

The data that support the findings of this study are available upon reasonable request.

## References

[1] El-Brolosy MA, Stainier DYR (2017). Genetic compensation: a phenomenon in search of mechanisms. PLoS Genet..

[2] Liu Y, Beyer A, Aebersold R (2016). On the dependency of cellular protein levels on mRNA abundance. Cell.

[3] Banaszynski LA, Chen L-c, Maynard-Smith LA, Ooi AGL, Wandless TJ (2006). A rapid, reversible, and tunable method to regulate protein function in living cells using synthetic small molecules. Cell.

[4] Armstrong CM, Goldberg DE (2007). An FKBP destabilization domain modulates protein levels in Plasmodium falciparum. Nat. Methods.

[5] Herm-Gotz A (2007). Rapid control of protein level in the apicomplexan Toxoplasma gondii. Nat. Methods.

[6] Maynard-Smith LA, Chen L-c, Banaszynski LA, Ooi AGL, Wandless TJ (2007). A directed approach for engineering conditional protein stability using biologically silent small molecules. J. Biol. Chem..

[7] Pratt MR, Schwartz EC, Muir TW (2007). Small-molecule-mediated rescue of protein function by an inducible proteolytic shunt. Proc. Natl Acad. Sci. USA.

[8] Nishimura K, Fukagawa T, Takisawa H, Kakimoto T, Kanemaki M (2009). An auxin-based degron system for the rapid depletion of proteins in nonplant cells. Nat. Methods.

[9] Neklesa TK (2011). Small-molecule hydrophobic tagging-induced degradation of HaloTag fusion proteins. Nat. Chem. Biol..

[10] Renicke C, Schuster D, Usherenko S, Essen L-O, Taxis C (2013). A LOV2 domain-based optogenetic tool to control protein degradation and cellular function. Chem. Biol..

[11] Bonger KM, Rakhit R, Payumo AY, Chen JK, Wandless TJ (2014). General method for regulating protein stability with light. ACS Chem. Biol..

[12] Hermann A, Liewald JF, Gottschalk A (2015). A photosensitive degron enables acute light-induced protein degradation in the nervous system. Curr. Biol..

[13] Rothbauer U (2006). Targeting and tracing antigens in live cells with fluorescent nanobodies. Nat. Methods.

[14] Caussinus E, Kanca O, Affolter M (2012). Fluorescent fusion protein knockout mediated by anti-GFP nanobody. Nat. Struct. Mol. Biol..

[15] Daniel K (2018). Conditional control of fluorescent protein degradation by an auxin-dependent nanobody. Nat. Commun..

[16] Clift D (2017). A method for the acute and rapid degradation of endogenous proteins. Cell.

[17] Chen X (2019). Degradation of endogenous proteins and generation of a null-like phenotype in zebrafish using Trim-Away technology. Genome Biol..

[18] Sakamoto KM (2001). Protacs: Chimeric molecules that target proteins to the Skp1–Cullin–F box complex for ubiquitination and degradation. Proc. Natl Acad. Sci. USA.

[19] Yin Q (2009). E2 interaction and dimerization in the crystal structure of TRAF6. Nat. Struct. Mol. Biol..

[20] Guntas G (2015). Engineering an improved light-induced dimer (iLID) for controlling the localization and activity of signaling proteins. Proc. Natl Acad. Sci. USA.

[21] Kennedy MJ (2010). Rapid blue-light-mediated induction of protein interactions in living cells. Nat. Methods.

[22] Mulholland CB (2015). A modular open platform for systematic functional studies under physiological conditions. Nucleic Acids Res..

[23] Liang F-S, Ho WQ, Crabtree GR (2011). Engineering the ABA plant stress pathway for regulation of induced proximity. Sci. Signal..

[24] Miyamoto T (2012). Rapid and orthogonal logic gating with a gibberellin-induced dimerization system. Nat. Chem. Biol..

[25] Banaszynski LA, Liu CW, Wandless TJ (2005). Characterization of the FKBP·rapamycin·FRB ternary complex. J. Am. Chem. Soc..

[26] Schwanhausser B (2011). Global quantification of mammalian gene expression control. Nature.

[27] Hansen S (2017). Design and applications of a clamp for Green Fluorescent Protein with picomolar affinity. Sci. Rep..

[28] Fridy PC (2014). A robust pipeline for rapid production of versatile nanobody repertoires. Nat. Methods.

[29] Sporbert A, Gahl A, Ankerhold R, Leonhardt H, Cardoso MC (2002). DNA polymerase clamp shows little turnover at established replication sites but sequential de novo assembly at adjacent origin clusters. Mol. Cell.

[30] Levin DS, McKenna AE, Motycka TA, Matsumoto Y, Tomkinson AE (2000). Interaction between PCNA and DNA ligase I is critical for joining of Okazaki fragments and long-patch base-excision repair. Curr. Biol..

[31] Mortusewicz O, Rothbauer U, Cardoso MC, Leonhardt H (2006). Differential recruitment of DNA Ligase I and III to DNA repair sites. Nucleic Acids Res..

[32] Conradt B, Wu YC, Xue D (2016). Programmed cell death during Caenorhabditis elegans development. Genetics.

[33] Horvitz HR (2003). Worms, life, and death (Nobel lecture). ChemBioChem.

[34] Schlehuber S, Skerra A (2002). Tuning ligand affinity, specificity, and folding stability of an engineered lipocalin variant—a so-called ‘anticalin’ - using a molecular random approach. Biophys. Chem..

[35] McMahon C (2018). Yeast surface display platform for rapid discovery of conformationally selective nanobodies. Nat. Struct. Mol. Biol..

[36] Tiede C (2017). Affimer proteins are versatile and renewable affinity reagents. eLife.

[37] Zimmermann I (2018). Synthetic single domain antibodies for the conformational trapping of membrane proteins. eLife.

[38] Gilbreth RN (2011). Isoform-specific monobody inhibitors of small ubiquitin-related modifiers engineered using structure-guided library design. Proc. Natl Acad. Sci. USA.

[39] Dreier B, Pluckthun A (2012). Rapid selection of high-affinity binders using ribosome display. Methods Mol. Biol..

[40] Moutel S (2016). NaLi-H1: A universal synthetic library of humanized nanobodies providing highly functional antibodies and intrabodies. eLife.

[41] Kirchhofer A (2009). Modulation of protein properties in living cells using nanobodies. Nat. Struct. Mol. Biol..

[42] Kummer L (2012). Structural and functional analysis of phosphorylation-specific binders of the kinase ERK from designed ankyrin repeat protein libraries. Proc. Natl Acad. Sci. USA.

[43] Koide A, Abbatiello S, Rothgery L, Koide S (2002). Probing protein conformational changes in living cells by using designer binding proteins: Application to the estrogen receptor. Proc. Natl Acad. Sci. USA.

[44] Striebel F, Imkamp F, Özcelik D, Weber-Ban E (2014). Pupylation as a signal for proteasomal degradation in bacteria. Biochim. Biophys. Acta.

[45] Bayle JH (2006). Rapamycin analogs with differential binding specificity permit orthogonal control of protein activity. Chem. Biol..

[46] Gao Y (2016). Complex transcriptional modulation with orthogonal and inducible dCas9 regulators. Nat. Methods.

[47] Erazo-Oliveras A (2014). Protein delivery into live cells by incubation with an endosomolytic agent. Nat. Methods.

[48] Herce HD (2017). Cell-permeable nanobodies for targeted immunolabelling and antigen manipulation in living cells. Nat. Chem..

[49] Tsukamoto T (2000). Visualization of gene activity in living cells. Nat. Cell Biol..

[50] Nabhan JF, Pan H, Lu Q (2010). Arrestin domain‐containing protein 3 recruits the NEDD4 E3 ligase to mediate ubiquitination of the β2‐adrenergic receptor. EMBO Rep..

[51] Wolf C (2016). RPA and Rad51 constitute a cell intrinsic mechanism to protect the cytosol from self DNA. Nat. Commun..

[52] Liu N (2013). Intrinsic and extrinsic connections of Tet3 dioxygenase with CXXC zinc finger modules. PLoS One.

[53] Chagin VO (2016). 4D Visualization of replication foci in mammalian cells corresponding to individual replicons. Nat. Commun..

[54] Chiu HY (2016). Intracellular chromobody delivery by mesoporous silica nanoparticles for antigen targeting and visualization in real time. Sci. Rep..

[55] Ran F (2013). Genome engineering using the CRISPR-Cas9 system. Nat. Protoc..

[56] Hermann M (2014). Binary recombinase systems for high-resolution conditional mutagenesis. Nucleic Acids Res..

[57] Ellis HM, Horvitz HR (1986). Genetic control of programmed cell death in the nematode C. elegans. Cell.

[58] Mello C, Fire A (1995). DNA transformation. Methods Cell Biol..

[59] Frøkjær-Jensen C (2014). Random and targeted transgene insertion in Caenorhabditis elegans using a modified Mos1 transposon. Nat. Methods.

[60] Zeilich, J., Mangal, S., Zanin, E. & Lambie, E. J. Establishment of a CRISPR/Cas9-based strategy for inducible protein dimerization. *microPublication Biology*. 10.17912/W2208R (2018). 10.17912/W2208R PMC7252319 32550367

[61] Wei H, Yan B, Gagneur J, Conradt B (2017). Caenorhabditis elegans CES-1 Snail represses pig-1 MELK expression to control asymmetric cell division. Genetics.

[62] Schwartz HT, Horvitz HR (2007). The C. elegans protein CEH-30 protects male-specific neurons from apoptosis independently of the Bcl-2 homolog CED-9. Genes Dev..

