# Supplementary material for: Tunable light and drug induced depletion of target proteins
Source: Nat Commun. 2020 Jan 16;11:304. doi: 10.1038/s41467-019-14160-8 (PMC6965615; doi:10.1038/s41467-019-14160-8)
Supplement: Supplementary file 1 — Supplementary Information [file 41467_2019_14160_MOESM1_ESM.pdf]

Supplementary information

Tunable light and drug induced depletion of target proteins

Deng *et al.*

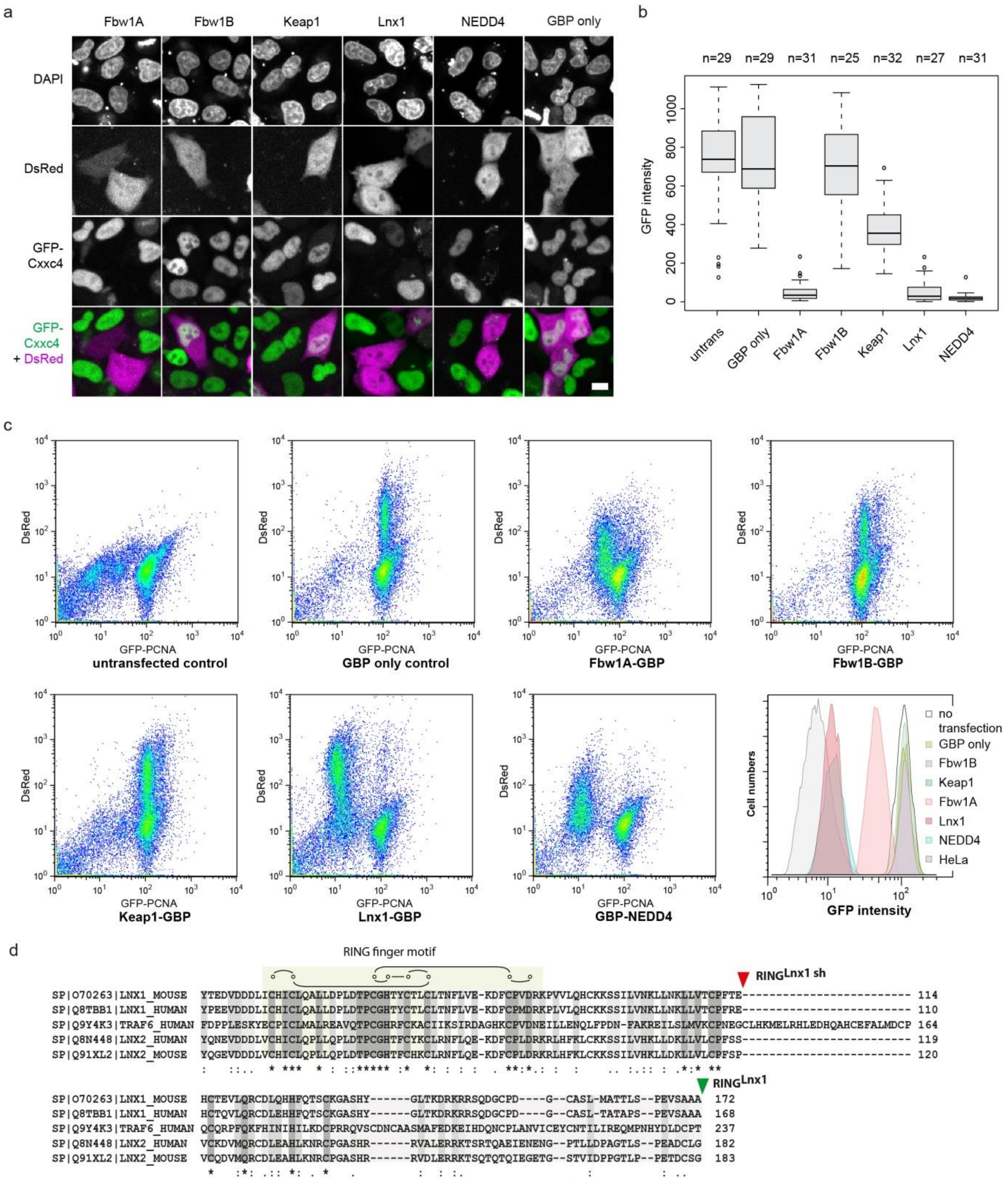

e

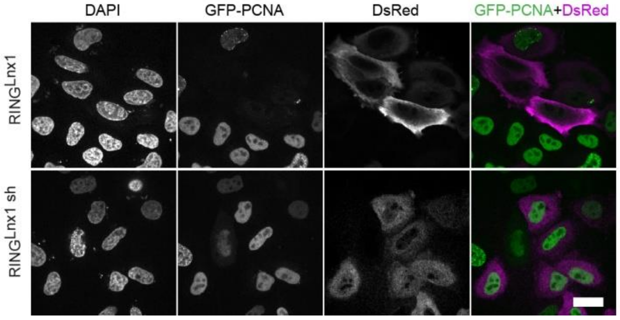

f

Protein	Efficiency	Fragments size (AAs)	Type of domain
Fbw1A	middle	239 (1-239)	F-box (Cullin-dependent)
Fbw1B	not work	228 (1-228)	F-box (Cullin-dependent)
Keap1	low	323 (1-323)	F-box (Cullin-dependent)
Lnx1	high	171 (1-171)	RING
NEDD4	high	283 (921-1303)	HECT

Supplementary Figure 1 Screening E3 ligase domains for efficiency in protein depletion.
a, depletion of GFP-CXXC4 with candidate E3 ligase domains fused with a GFP binding nanobody (GBP1). DsRed expression is linked via an IRES with expression of the respective E3 ligase fusion to mark cells transiently expressing the E3 ligase fusion. Scale bar represents 10 µm. **b,** Image analysis based quantification of GFP-CXXC4 fluorescence in cells expressing the different E3 ligase fusions. Boxes show the 25th to 75th percentile range (interquartile range, IQR), and the whiskers indicate 1.5 times IQR, outliers are the values higher than 1.5 times IQR above the third quartile or values lower than 1.5 times IQR below the first quartile, medians are shown as lines in the boxes. **c,** Flow cytometry profiles of GFP-PCNA HeLa cells expressing the five different E3 ligase nanobody fusions in comparison to nanobody only or untransfected GFP-PCNA HeLa cells and to HeLa wild-type cells. The GFP-PCNA intensity of (DsRed positive) cells are displayed in the histogram. **d,** Protein sequence alignment of RING domains from closely related LNX proteins and TRAF6. **e,** The efficiency in protein depletion of the long RING ($RING^{Lnx1}$) and short RING ($RING^{Lnx1\ sh}$) fragments was compared as in part **a**. Scale bar is 20 µm. **f,** Features list of the E3 ligases tested in this study.

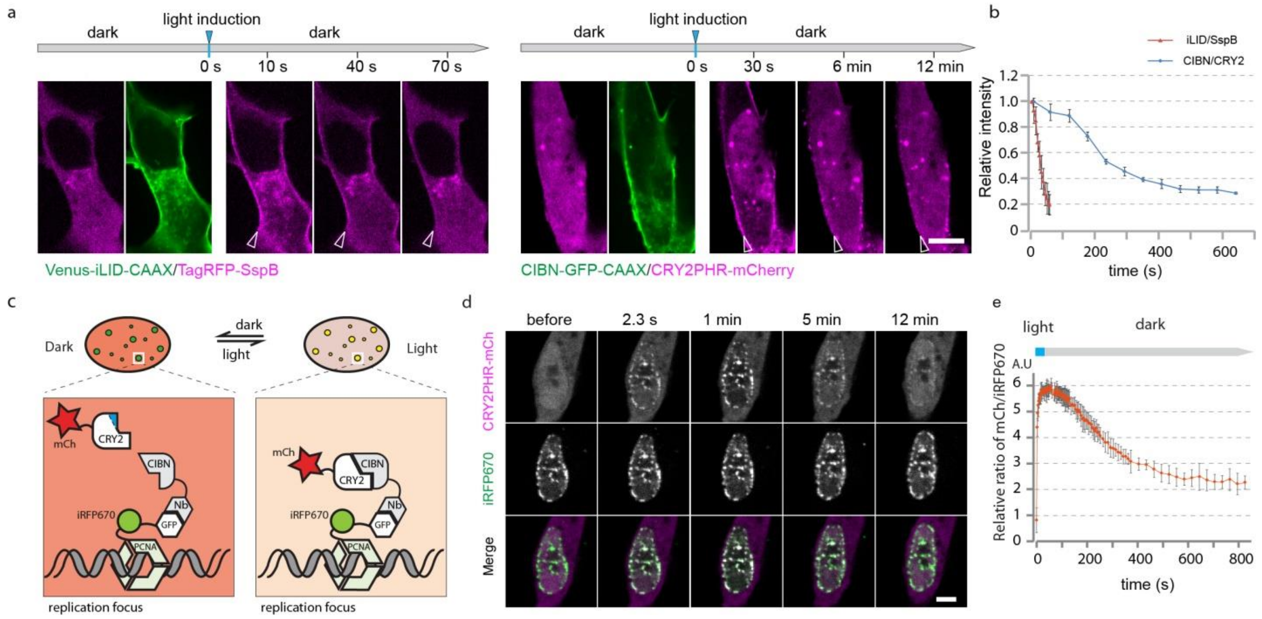

Supplementary Figure 2 Association and dissociation dynamics of light induced heterodimer components.

a, Two LID pairs, iLID/SspB and CIBN/CRY2PHR, were tagged with different fluorescent proteins and tested by live cell imaging. One component of each LID heterodimer (iLID and CIBN) was anchored at the cell membrane by fusion with the K-RAS4b CAAX isoprenylation sequence (KKKKKKSKTKCVIM) at the C-termini of the two proteins. Light induced heterodimer formation at the cell membrane (marked with arrows) was monitored by fluorescence microscopy; please note the different time intervals indicated above. **b,** The heterodimer formation was quantified as enrichment of the second component (TagRFP-SspB and CRY2PHR-mCherry) at the membrane over time. The direct comparison shows that the CIBN/CRY2PHR heterodimer has a longer half-time (about 5 min) and was therefore chosen for subsequent experiments. For each group, five cells were measured, and results are shown as mean value ± SD. The CIBN/CRY2PHR heterodimer formation was also tested with another cellular anchor as outlined in **c**. Here, the CIBN part was recruited to replication foci via a GFP binding nanobody (GBP1) that binds the GFP-PCNA fusion protein, which was in addition fused with near-infrared FP iRFP670 for imaging. **d,** The enrichment of CRY2PHR-mCh at replication foci after light induced dimerization was monitored with fluorescence microscopy over time. **e,** Quantification of enrichment at replication foci reveals a similar dissociation dynamics as for the membrane recruitment in **b** with half-times of about 5 min. Error bars are defined as SD., number of analyzed cells = 9. For **a** - **e**, Heterodimer formation was induced with a 488 nm laser (10% of 2.5 mW for 0.6 s); scale bars represent 10 µm.

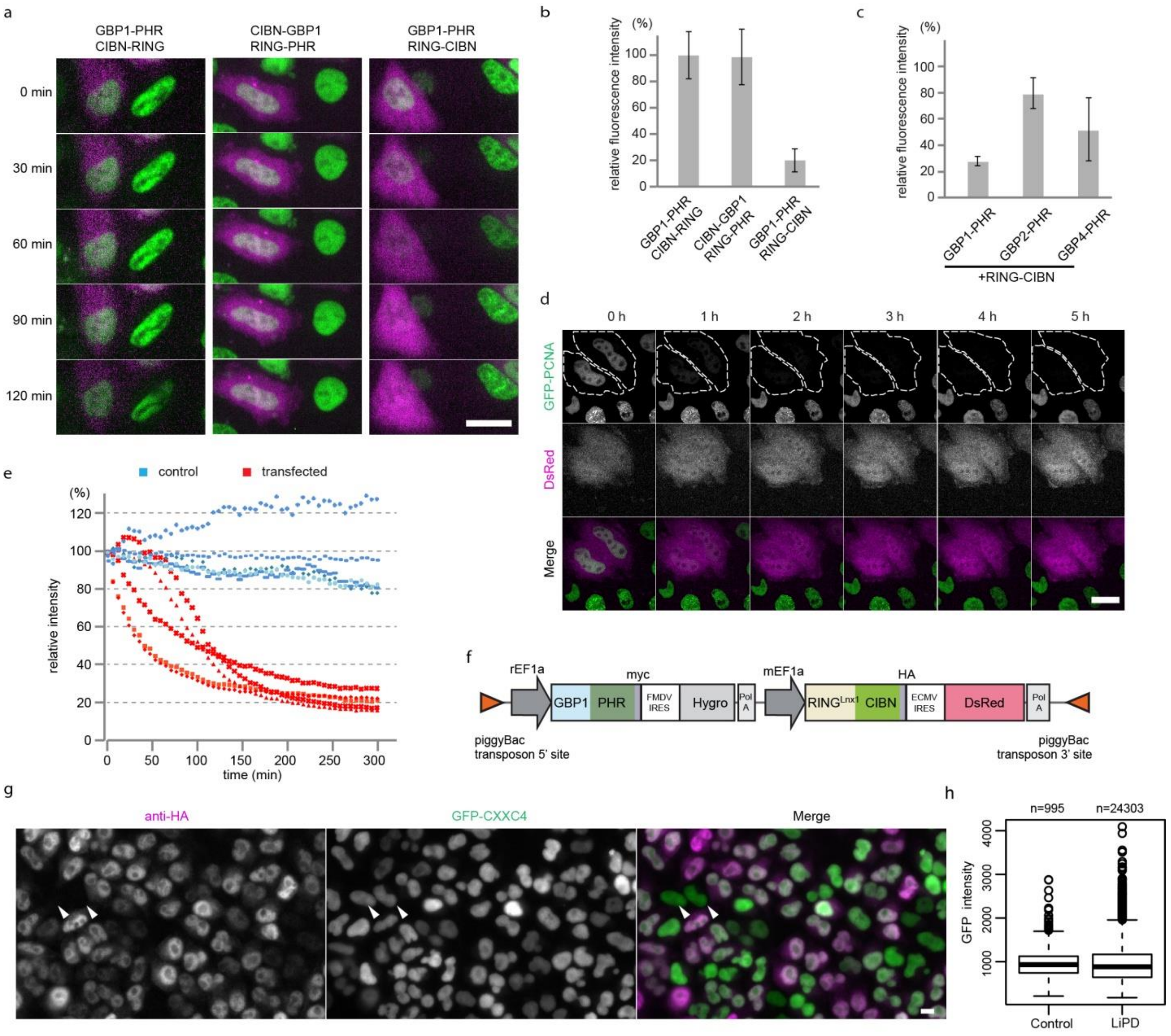

Supplementary Figure 3 Optimization of light induced protein depletion (LiPD).

a, For LiPD the more stable CIBN/PHRCRY2 heterodimer pair (see Supplementary Fig. 2) was chosen and different orientations for the functional domain fusions were tested. We compared N- and C-terminal fusions with the targeting nanobody (GBP1) and the RING ubiquitin ligase domain (RING). Cells expressing the LiPD construct were identified by the co-expressed DsRed marker shown in magenta. The capacity of these different LiPD constructs to deplete a cellular GFP-tagged protein (GFP-PCNA stably expressed in HeLa cells) was monitored over 120 min with repeated light induced dimerization using a 488 nm laser (10% of 2.5 mW for 0.6 s) every 10 min. **b,** The efficiency of different LiPD constructs to deplete GFP-PCNA was evaluated at the 120 min time point by comparing the average nuclear fluorescence intensity at 0 min and 120 min. The direct comparison shows that the GBP1-PHR/RING-CIBN combination was by far the most efficient in depleting the cellular target protein. Error bars are defined as SD. **c,** Two more GFP binding nanobodies (GBP2 and GBP4) were tested using this LiPD orientation and the same experimental set-up but found to be less efficient. Error bars are defined as

SD. **d,** The optimized LiPD construct (GBP1-PHR/RING-CIBN) was used to deplete the stably expressed GFP-PCNA and cells were monitored over 5 h. Cells expressing the LiPD construct were identified by the co-expressed DsRed and are marked with a dashed line. **e,** Relative nuclear fluorescence intensities of five cells for each group were measured every 10 min and plotted. **f,** The optimal LiPD combination (GBP1-PHR/RING-CIBN) identified in this study was placed in a piggyBac vector for further applications (see Fig. 1 and Supplementary Fig. 4). A single two promoter cassette was generated for constant stoichiometric expression of LiPD system components including a drug resistance gene (hygro, hygromycin B phosphotransferase) for selection and a fluorescent marker (DsRed) to identify cells expressing the LiPD components. Transposon 5' and 3' inverted terminal repeats (ITRs) are incorporated enabling highly efficient piggyBac transposase mediated cell line generation. **g,** Images of GFP-CXXC4 cells stably expressing the LiPD system. GFP-CXXC4 cells expressing the LiPD system (anti-HA positive cells) and control cells without the LiPD (arrowhead, anti-HA negative) were mixed and imaged for detection of background protein depletion caused by the system under these experimental conditions. **h,** GFP intensities of cells with and without the LiPD were quantified and plotted, showing on average only a weak (~3%) background degradation of the target protein GFP-CXXC4. Boxes show the 25th to 75th percentile range (interquartile range, IQR), and the whiskers indicate 1.5 times IQR, outliers are the values higher than 1.5 times IQR above the third quartile or values lower than 1.5 times IQR below the first quartile, medians are shown as lines in the boxes. Scale bars stand for 10 µm.

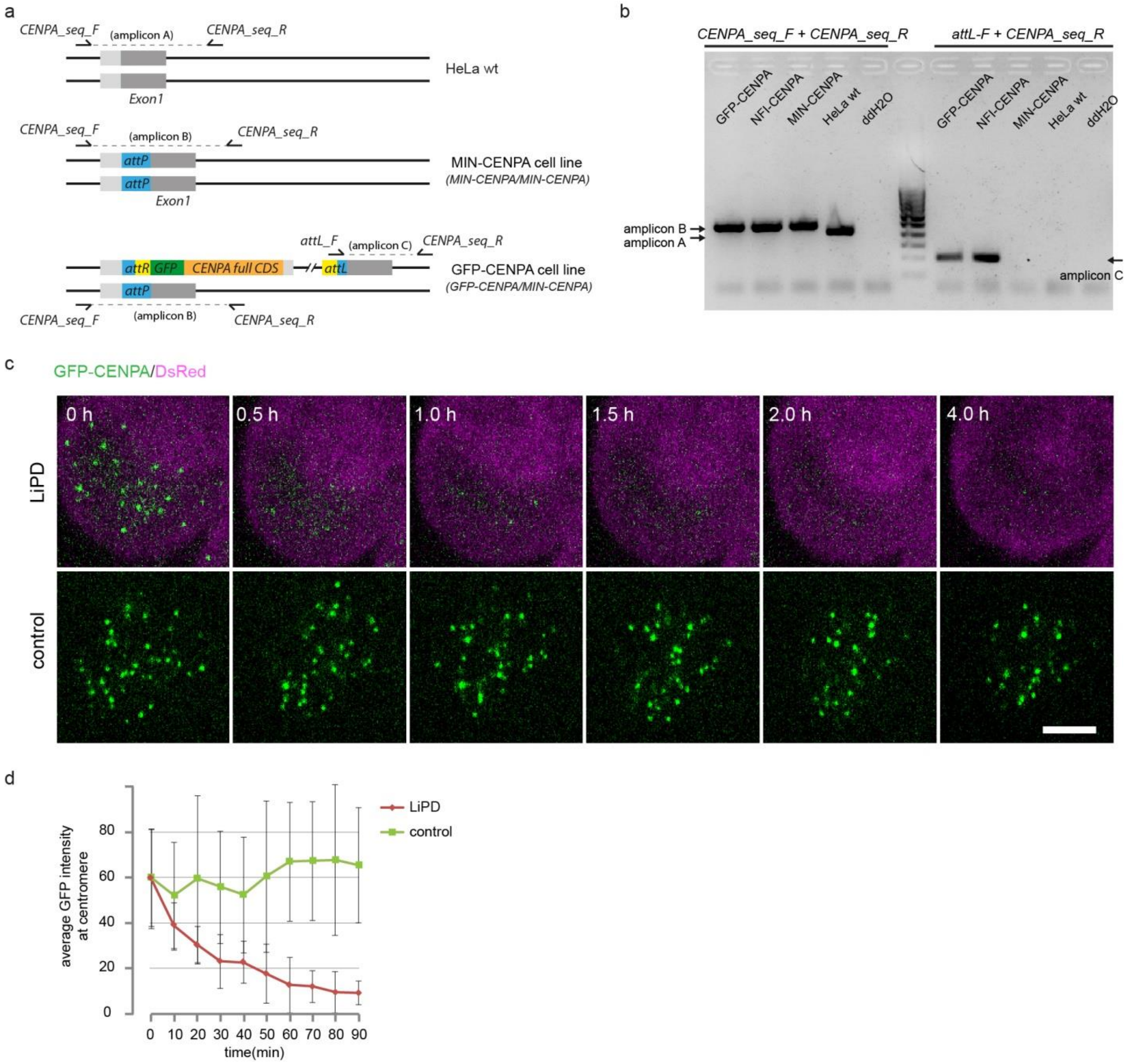

Supplementary Figure 4 Light induced depletion of tagged endogenous proteins.

a, A GFP-CENPA knock-in cell line was generated using a two step protocol. First, *attP* (MIN-tag) sites were introduced using CRISPR/Cas9 at the 5' end of the *CENPA* ORF after the start codon (MIN-CENPA). Second, the *GFP-CENPA* was integrated into the *attP* site by the Bxb1 mediated recombination and thereby placed under the transcriptional control of the endogenous *CENPA* promoter (GFP-CENPA). **b,** Characterization of the GFP-CENPA endogenous knock-in cell line by PCR. Two pairs of primers as shown in **a** were used to amplify the recombinant *GFP-CENPA* locus, one primer pair detects the *attP* site inserted at the *CENPA* locus (amplicon B), while the other pair detects the Bxb1 mediated insertion (*attL*) of GFP-CENPA at the locus (amplicon C). Both primer pairs together indicate that the GFP-CENPA cell line is heterozygotic with one allele containing the GFP-CENPA knock-in and the other allele harboring the original MIN-tag at the endogenous *CENPA* locus. HeLa wt cells were used as negative control and heterozygotic NFI-CENPA (N-terminally FLAG-tagged CENPA) as positive control for amplicon C. **c,** Light induced depletion of GFP-CENPA by the LiPD system. GFP-CENPA was degraded quickly after

exposure to blue light (same protocol as in Supplementary Fig. 3a). Scale bar is 10 µm. **d,** Quantification of GFP-CENPA at centromeres after light induction. For each time point 26 to 57 centromeres were identified and measured. Average GFP intensity at recognized centromere is shown; error bars are defined as SD. However, as progressing protein depletion reduced the fluorescent label and left less recognizable centromeres we systematically underestimated the overall protein depletion.

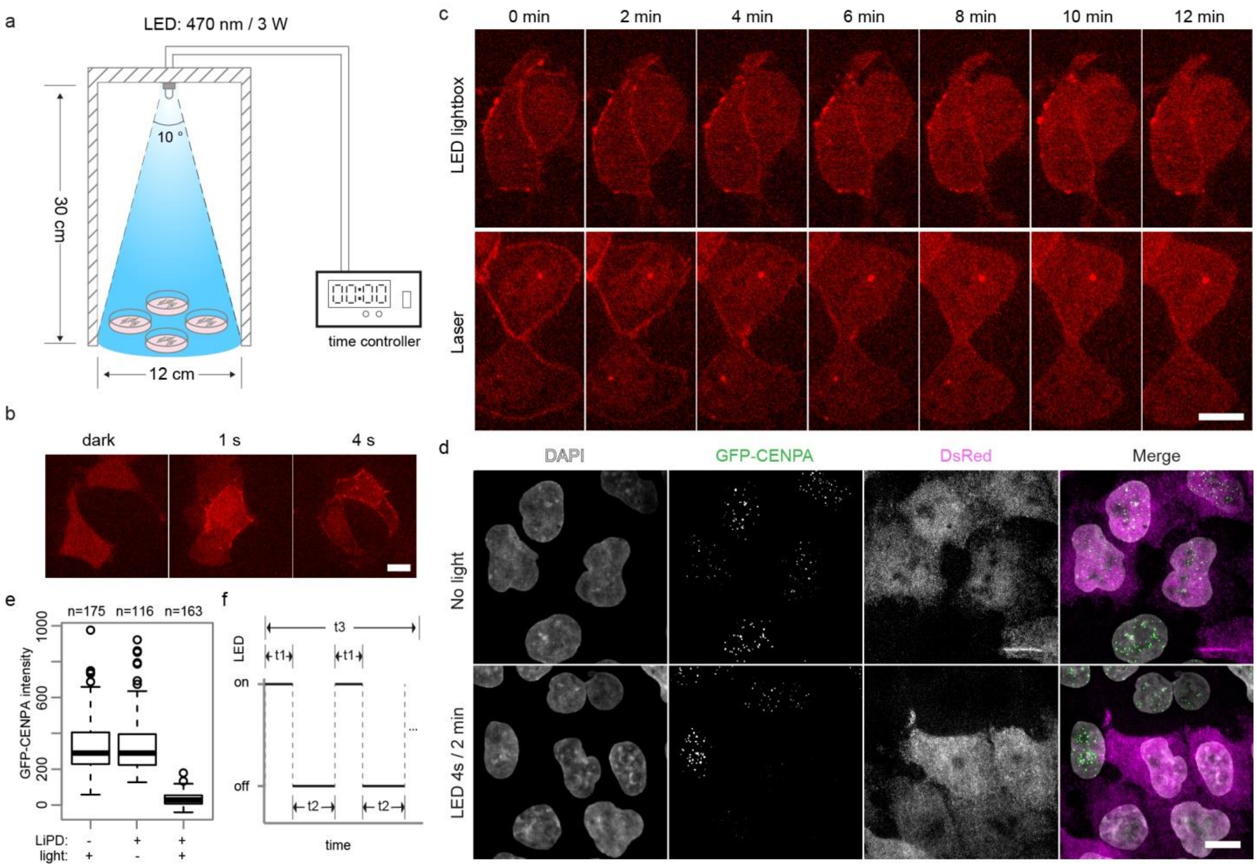

Supplementary Figure 5 Population-wide light induced protein depletion.

a, Schematic outline of the LED lightbox. **b,** LED light induced hetero-dimerization of Venus-CIBN-CAAX and mCh-CRY2PHR. LED lightbox induced heterodimerization of mCherry tagged CRY2PHR with Venus-CIBN-CAAX at the cell membrane following 1 or 4 s of illumination. **c,** Heterodimers as in **b** were induced with laser (488 nm, 10% of 2.5 mW for 0.6 s) and LED lightbox (4 s). The dissociation of heterodimers was monitored over time revealing a similar half life time of about 6 min for LED and laser induced dimers. **d,** Stable LiPD/GFP-CENPA cells (DsRed expressing) were mixed with GFP-CENPA HeLa cells (serving as internal controls) and examined for LED light induced depletion of GFP-CENPA. After 4 h no GFP-CENPA signal was detected in LED illuminated cells expressing the LiPD (DsRed positive shown in magenta) while little to no depletion was observed in neighboring cells without the LiPD or in cells that were not illuminated (no light). Scale bars represent 10 µm. **e,** Quantification of the LED light induced depletion of GFP-CENPA in cells. Boxes show the 25th to 75th percentile range (interquartile range, IQR), and the whiskers indicate 1.5 times IQR, outliers are the values higher than 1.5 times IQR above the third quartile or values lower than 1.5 times IQR below the first quartile, medians are shown as lines in the boxes. **f,** Scheme of the binary program for light induced dimerization with the LED lightbox. In this set-up the duration of the illumination periods (t1), the dark intervals (t2) and the entire process (t3) can be defined and optimized. Scale bars are 10 µm.

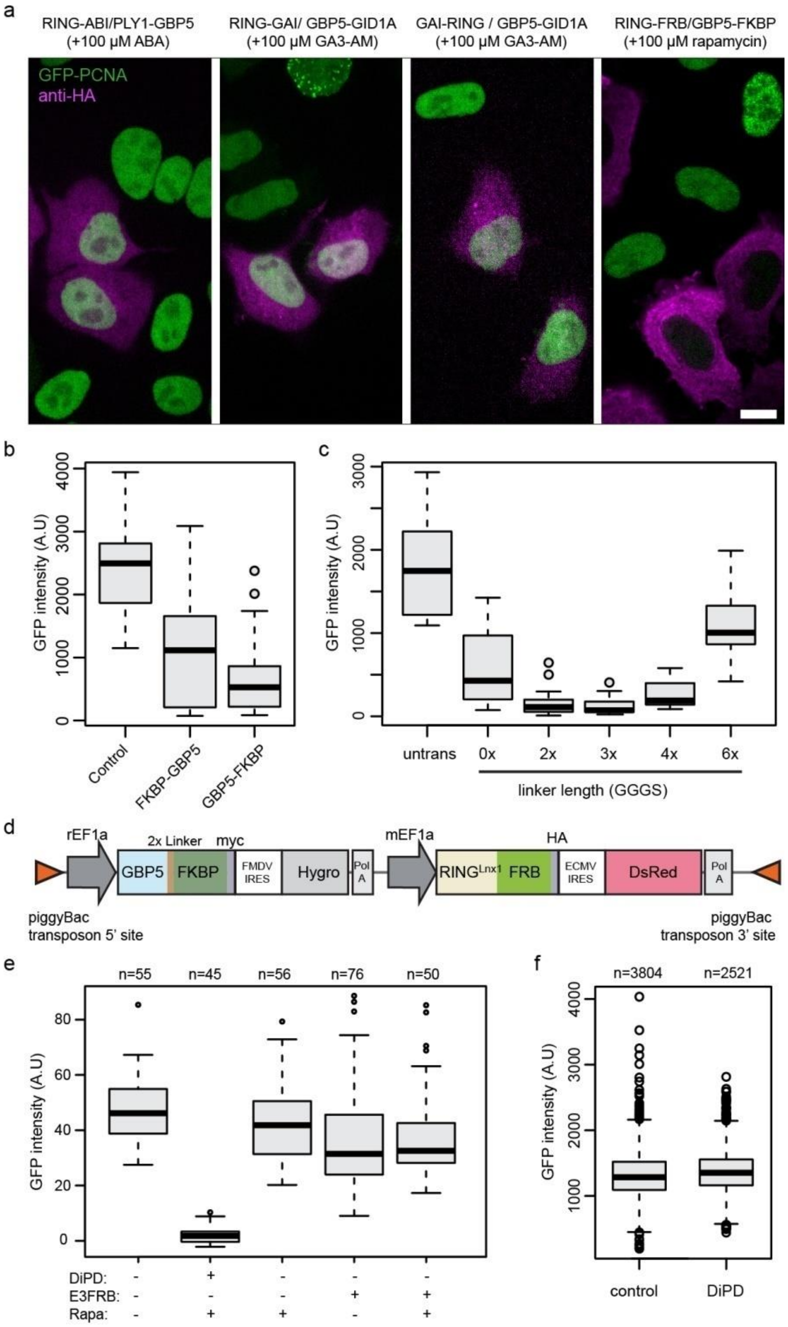

Supplementary Figure 6 Optimization of the drug induced protein depletion (DiPD) system.

a, Representative fluorescent images of directed protein depletion by three different chemically induced protein interaction pairs. Among the tested combinations, only the FKBP-FRB pair, and not the ABI-PLY1 or GAI-GID1A systems, showed efficient depletion of the POI (GFP-PCNA, in green) after treatment with the corresponding inducers. The expression of the respective system was detected with antibodies recognizing the HA tag (in magenta). Scale bar represents 10 µm. **b,** Testing different GBP/FKBP orientations for the DiPD. GBP5 at both, the N- and C- terminus, of the FKBP unit reduced GFP-PCNA levels upon induction, whereby the N-terminal GBP5 fusion of FKBP showed a better efficiency. **c,** Effects

of the flexible linker on the efficiency of the DiPD system. Linkers with different length were tested for their effect on the drug induced depletion of GFP-PCNA protein. While DiPD constructs with the 2× and 3× GGGS linkers showed the best depletion efficiencies, the longer 6× GGGS linker seemed to impair protein depletion. The experiments were repeated with biologically independent samples (cell cultures): untransfected control (n = 13), 2× linker (n = 17), 3× linker (n = 16), 4× linker (n = 15), 6× linker (n = 14). Arbitrary units of GFP fluorescence are shown in box plots. **d,** Outline of the DiPD cassette compatible with piggyBac transposon system for stable genome integration. **e,** Control for E3 ligase expression and rapamycin treatment. GFP-PCNA cells were transfected to express either DiPD or E3FRB and treated with or without rapamycin. The comparison shows that all three components are required for efficient protein depletion. **f.** GFP-PCNA intensity is not affected by stable expression of the DiPD in the absence of rapamycin. The GFP-PCNA intensities of cells expressing (DiPD) or without DiPD (control) were quantified by high throughput imaging analysis. (**b, c, e** and **f**) Boxes show the 25th to 75th percentile range (interquartile range, IQR), and the whiskers indicate 1.5 times IQR, outliers are the values higher than 1.5 times IQR above the third quartile or values lower than 1.5 times IQR below the first quartile, medians are shown as lines in the boxes.

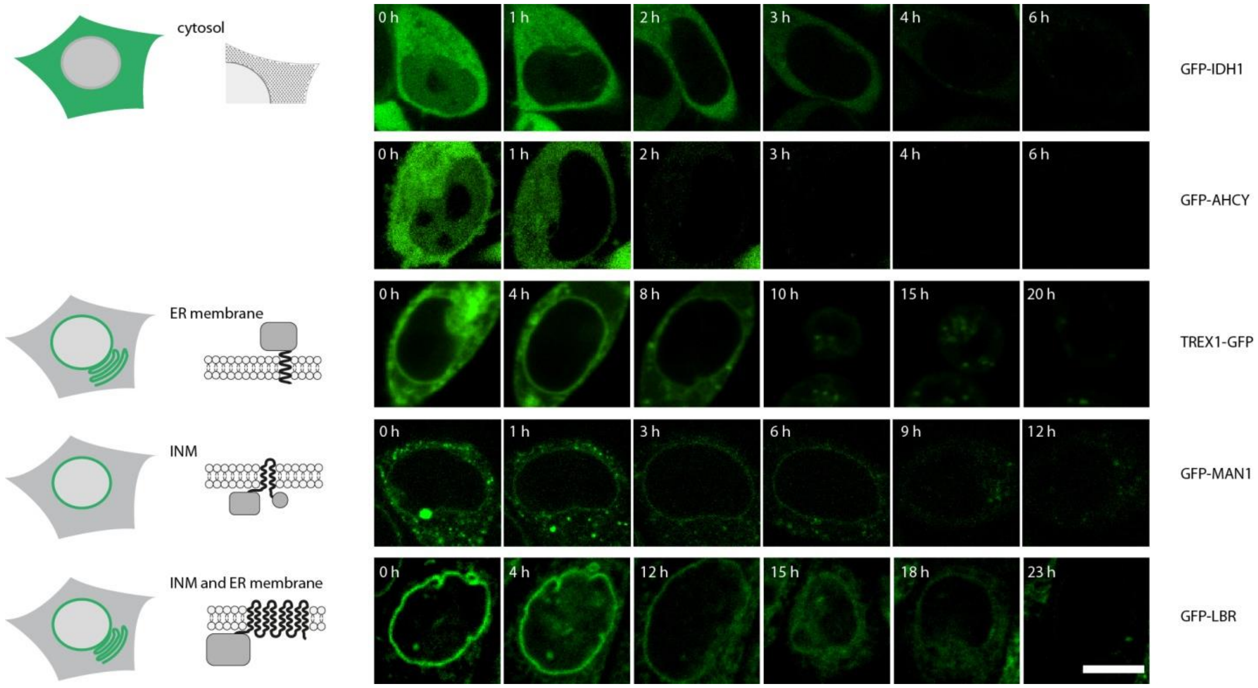

Supplementary Figure 7   Depletion of cytoplasmic and transmembrane proteins with the DiPD system.

Cytosolic proteins, IDH1 and AHCY (first two rows), were rapidly depleted with the DiPD system. Transmembrane proteins with single (TREX1), double (MAN1) or multiple (LBR) transmembrane helixes were fused to GFP, and depletion of the GFP fusions by the DiPD system was induced by the addition of rapamycin (last three rows). ER, endoplasmic reticulum; INM, inner nuclear membrane. Scale bar stands for 10 μm.

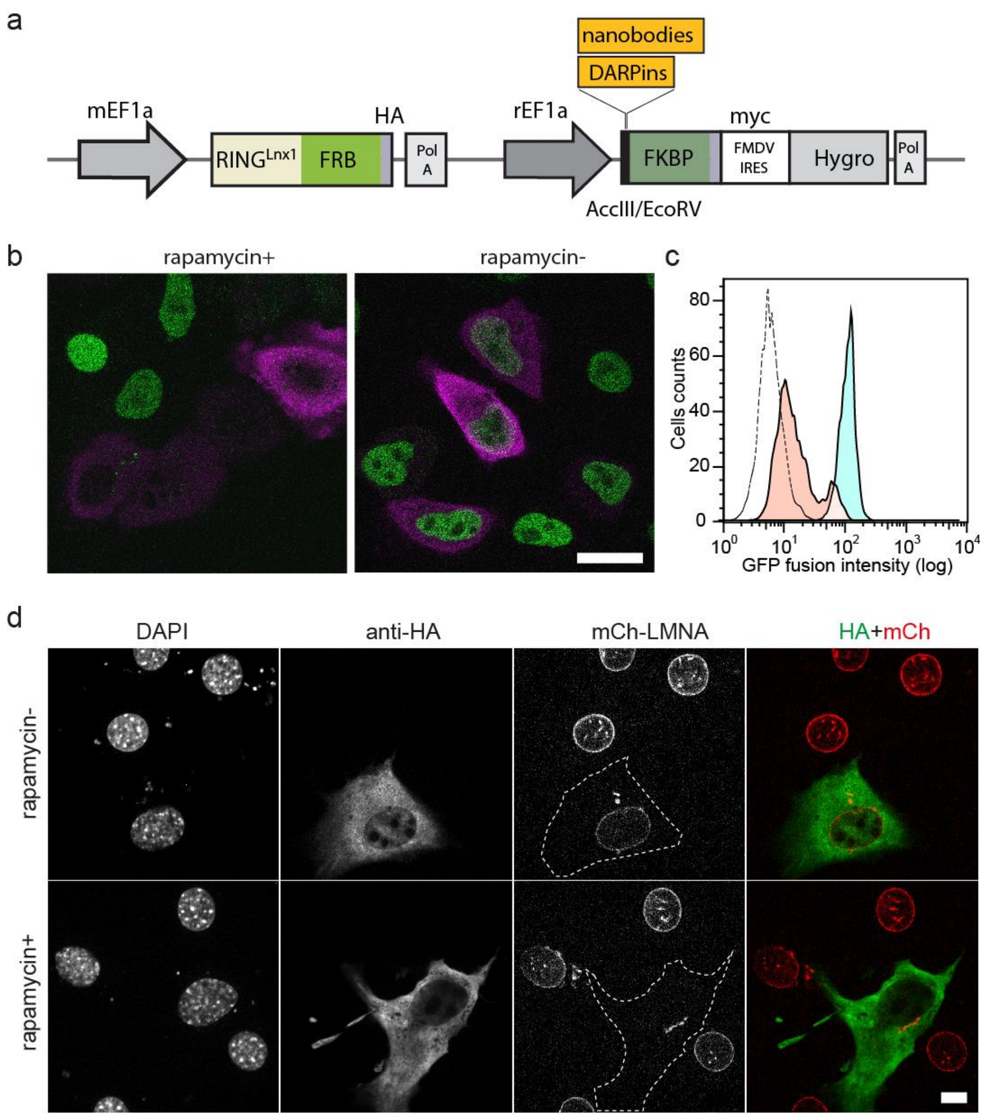

Supplementary Figure 8 Generalization of the DiPD system with different targeting modules.
a, Schematic outline of the DiPD vector accommodating different types of binding domains. To widen the application of the DiPD, a GFP binding DARPin and mCherry binding nanobody were used. **b,** GFP-PCNA was depleted inducibly in cells transfected with the DARPin based DiPD vector (in magenta). **c,** GFP-PCNA fluorescence in cells expressing the DARPin based DiPD was analyzed by flow-cytometry. Untreated cells showed a clear fluorescence signal (in blue) compared to wt HeLa cells (transparent peak with dashed line). Rapamycin treatment caused a roughly tenfold reduction in mean fluorescence distribution (in brown). For each group, about 10,000 cells were analyzed. **d,** Depletion of mCherry fusion protein with an mCherry binding nanobody by the DiPD system. MEF cells stably expressing mCherry tagged LaminA (mCh-LMNA) were transfected with the mCherry binding nanobody DiPD construct (marked with dashed line). The mCh-LMNA protein was efficiently depleted upon rapamycin induction. Scale bars are 10 µm.

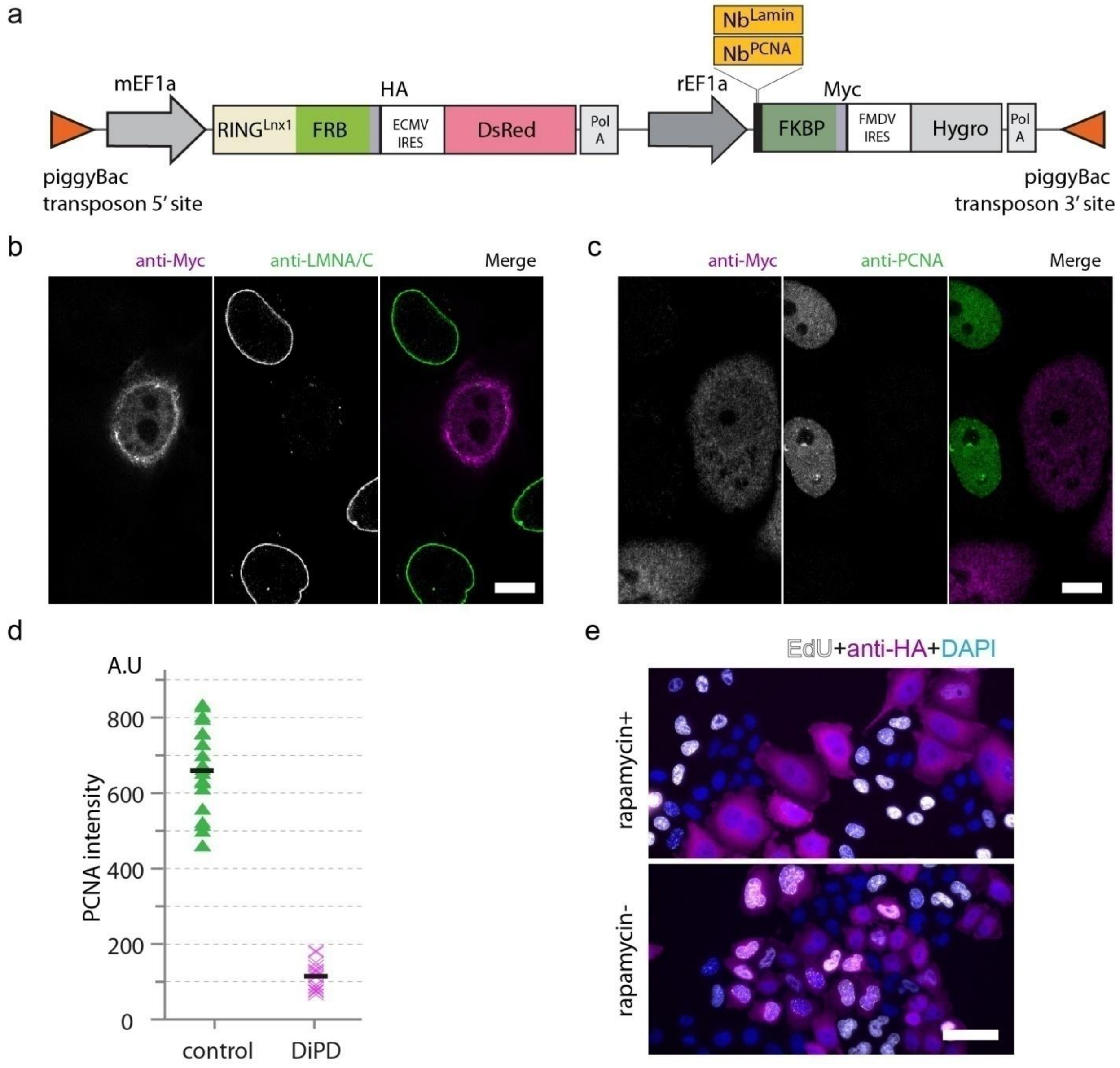

Supplementary Figure 9 Depletion of endogenous LMNA/C and PCNA with the DiPD system.
a, Schematic outline of LaminA and PCNA nanobodies (Nb) mediated DiPD for depletion of endogenous proteins. **b,c,** Depletion of endogenous LMNA/C (**b**) and PCNA (**c**) in HeLa cells with the stably integrated DiPD system. Endogenous proteins (detected by antibody) were depleted by the DiPD system (identified by anti-myc antibody) in the presence of rapamycin, but not in the co-cultured cells without the DiPD. Scale bar is 10 µm. **d,** Quantification of PCNA in cells with (DiPD) and without (control) the nanobody mediated DiPD (control group: n=22; and DiPD cells: n=17). **e,** No nuclear EdU labeling was detected in PCNA depleted cells (upper panel, rapamycin induced and DiPD expressing cells were identified with anti-HA staining in magenta). Without rapamycin induction (lower panel) EdU labeling was detected in cells independent of presence or absence of the DiPD system. Quantification is shown in Fig. 3a. Scale bar represents 50 µm.

a

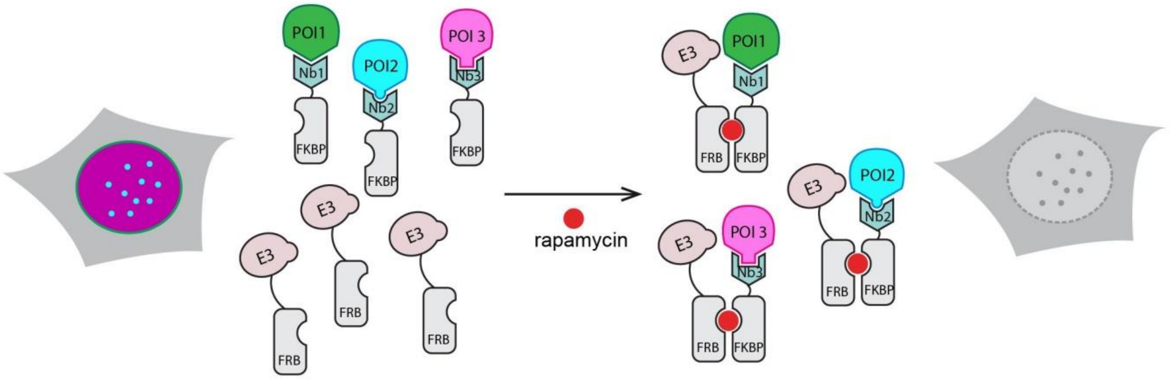

b

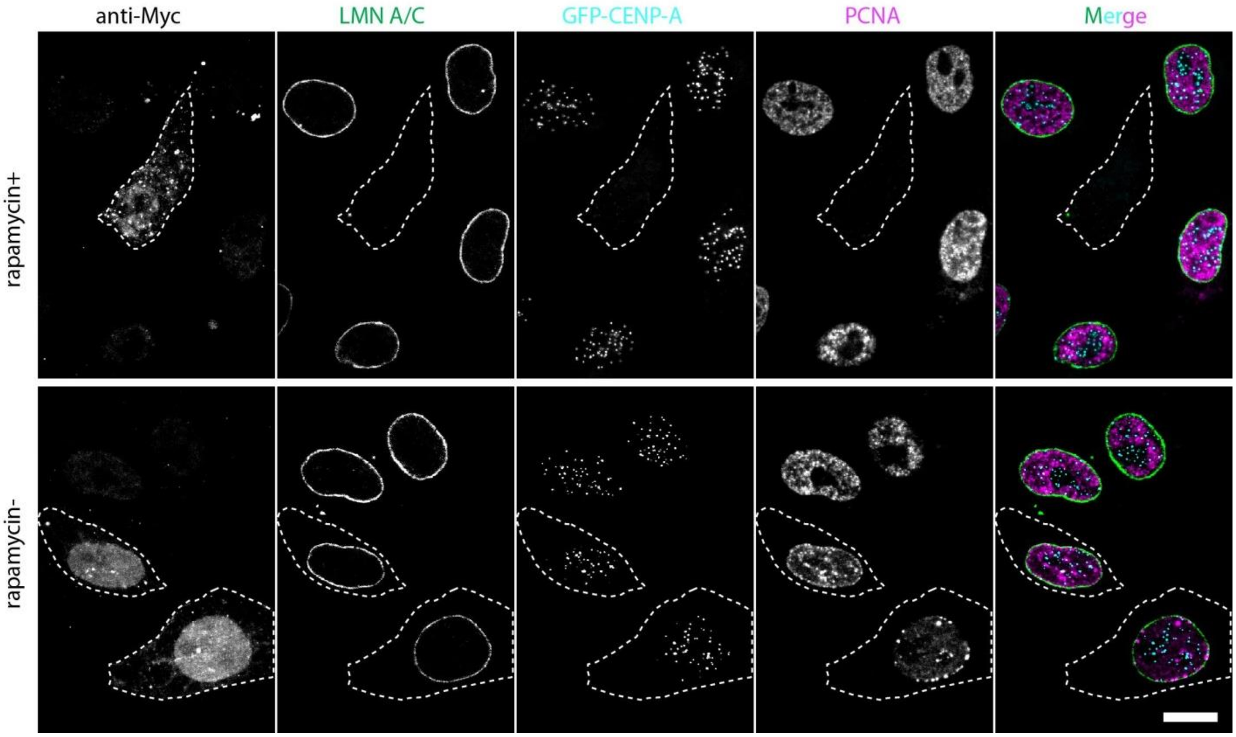

Supplementary Figure 10 Simultaneous depletion of multiple proteins by the DiPD system.

a, Schematic outline of the multi-protein targeting DiPD system. Multiple POIs were targeted by different protein specific targeting modules (Nbs) which were embedded in the DiPD system. Upon induction with rapamycin the POIs are ubiquitinated and degraded. **b,** Triple depletion of endogenous LMNA/C, PCNA and GFP-CENPA proteins. GFP-CENPA cells were transfected with DiPD constructs targeting LMNA/C, PCNA and GFP. Proteins were detected by immunostaining or GFP fluorescence after rapamycin treatment. A triple depletion of targeted proteins could be observed in the presence of rapamycin (upper panel), but not in cells without rapamycin induction (lower panel). Scale bar represents 10 µm.

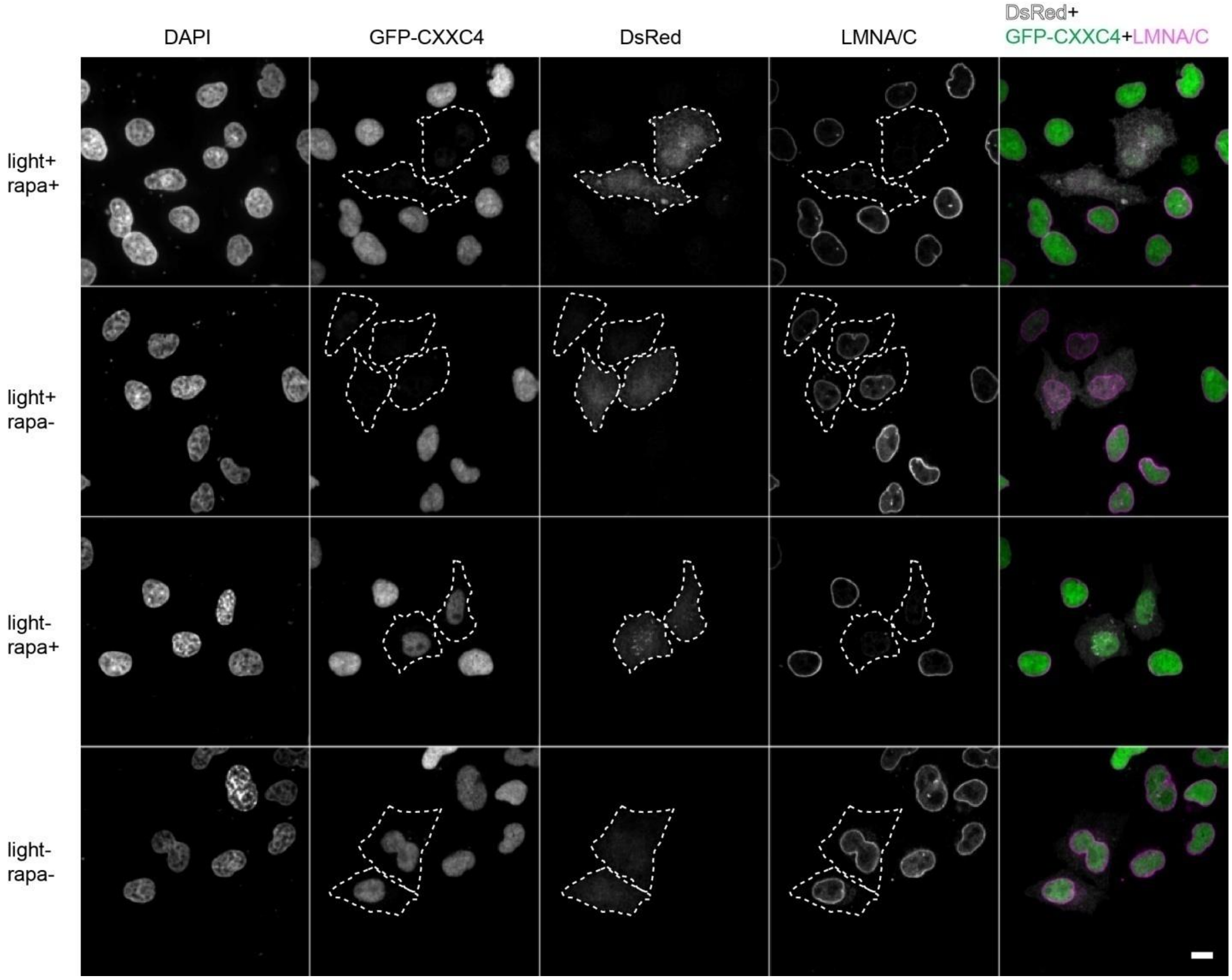

Supplementary Figure 11   Combinatorial depletion of two different proteins.

Combinatorial depletion of a nucleoplasmic (GFP-CXXC4) and a nuclear envelope protein (LMNA/C). Cells with the light- and drug- inducible protein depletion tools express DsRed as marker while surrounding DsRed negative cells serve as negative control. Scale bar is 10 µm.

a

```
Mouse       1   -MALKRIHKELNDLARDPPAQCSAGPVGDDMFHWQATIMGPNDSPYQGGVFFLTIHFPTDYPFKPPKVAFTTRIY  74
Human       1   -MALKRIHKELNDLARDPPAQCSAGPVGDDMFHWQATIMGPNDSPYQGGVFFLTIHFPTDYPFKPPKVAFTTRIY  74
Worm        1   -MALKRIQKELQDLGRDPPAQCSAGPVGDDLFHWQATIMGPPESPYQGGVFFLTIHFPTDYPFKPPKVAFTTRIY  74
Fruit fly   1   -MALKRINKELQDLGRDPPAQCSAGPVGDDLFHWQATIMGPPDSPYQGGVFFLTIHFPTDYPFKPPKVAFTTRIY  74
Yeast       1   MSSSKRIAKELSDLERDPPTSCSAGPVGDDLYHWQASIMGPADSPYAGGVFFLSIHFPTDYPFKPPKISFTTKIY  75
                  : *** ***.** ****:.*********::****:**** :*** ******:*************::***:**

Mouse      75   HPNINSNGSICLDILRSQWSPALTISKVLLSICSLLCDPNPDDPLVPEIARIYKTDREKYNRIAREWTQKYAM   147
Human      75   HPNINSNGSICLDILRSQWSPALTISKVLLSICSLLCDPNPDDPLVPEIARIYKTDREKYNRIAREWTQKYAM   147
Worm       75   HPNINSNGSICLDILRSQWSPALTISKVLLSICSLLCDPNPDDPLVPEIARIYKTDRERYNQLAREWTQKYAM   147
Fruit fly  75   HPNINSNGSICLDILRSQWSPALTISKVLLSICSLLCDPNPDDPLVPEIARIYKTDREKYNELAREWTRKYAM   147
Yeast      76   HPNINANGNICLDILKDQWSPALTLSKVLLSICSLLTDANPDDPLVPEIAHIYKTDRPKYEATAREWTKKYAV   148
                *****:**.******:.*******:*********** * ***********:****** :*:  *****:***:
```

b

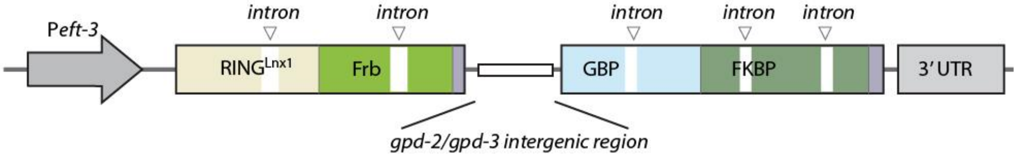

Supplementary Figure 12 Application of the DiPD system in *C. elegans*.

a, Alignment of the E2 ubiquitin conjugation enzyme UBE2D2 homologs shows high sequence conservation, which indicates a similar mechanism for protein ubiquitination and suggests that the mouse derived E3 RING^{Lnx1} ligase should function with the conserved *C. elegans* E2 enzyme. Protein sequences were taken from UniProt database (sequence IDs are: P62838 (mouse); P62837 (human); P35129 (worm); P25867 (fruit fly); P15731 (yeast)). **b,** Construction of a DiPD vector for expression in *C. elegans*. The DiPD system was codon-optimized for expression in *C. elegans* and placed under the transcriptional control of the ubiquitous *eft-3* promoter. Artificial introns were used to enhance the expression and a gpd-2/gpd-3 intergenic region was inserted to achieve equal expression levels of both parts.

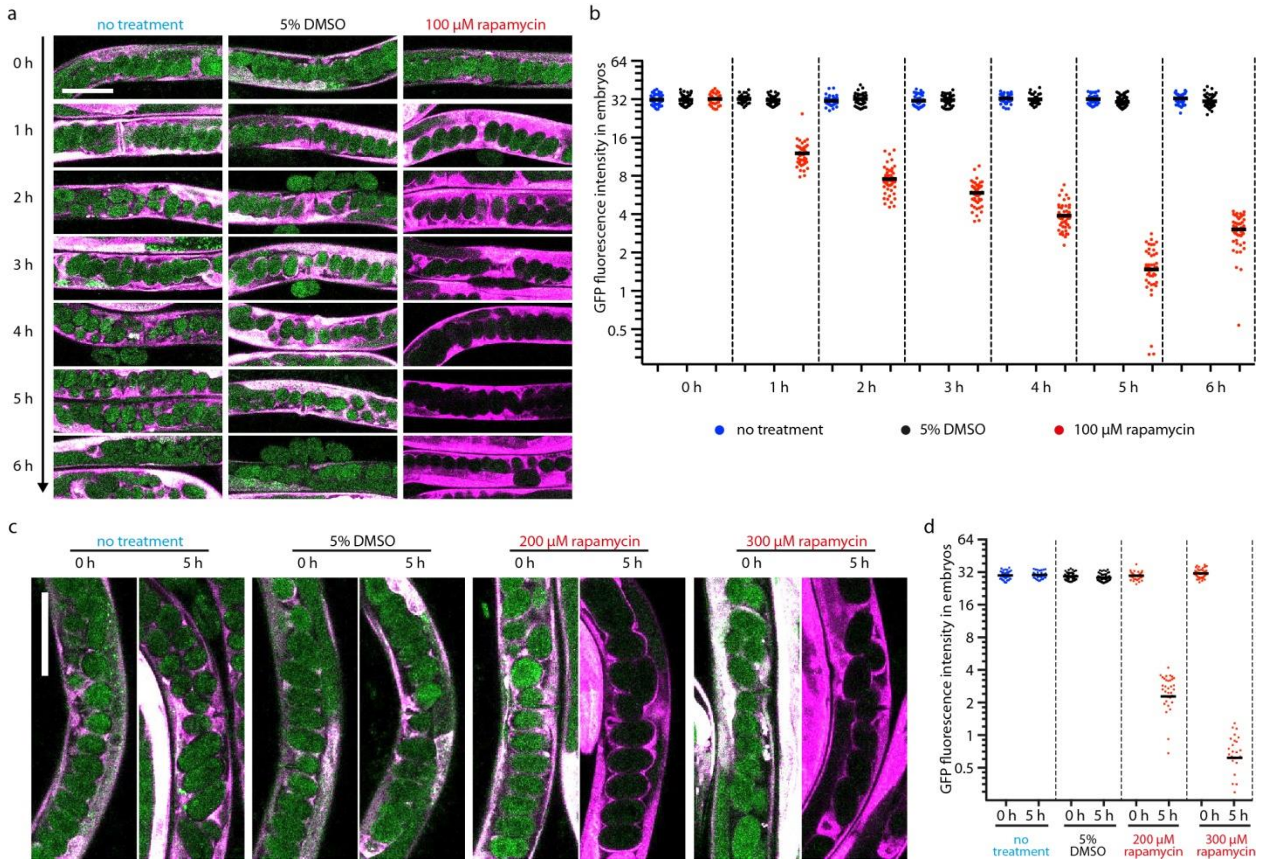

Supplementary Fig. 13 Time and dose dependent depletion of CED3::GFP in worm embryos.
a, Images of CED3::GFP in *C. elegans* embryos for the first 6 h after rapamycin induction. Untreated or DMSO treated worms serve as control. **b,** Quantitative analysis of CED3::GFP in worm embryos. Depletion of CED3::GFP could already be detected at the first time point (1h), and reached maximal depletion between 4 to 6 h. For each time point, 29 to 53 embryos were quantified. **c, d,** Rapamycin dose dependent depletion of the CED3::GFP in worms. Compared to **a** and **b**, two higher concentrations of rapamycin (200 µM and 300 µM) were administrated to worms, and the CED3::GFP in worm embryos was imaged (**c**) and analyzed (**d**) 5 h after treatment, showing a dose dependent depletion of the target protein. For each time point, 31 to 53 embryos were quantified. Scale bar is 50 µm.

Supplementary Table 1. Primers used in this study.

name	sequences
Fbxw1A-F	AAGCTAGCATGGACCCGGCAGAGG
Fbxw1A-R	CTAGATCTCACTCCGGCAGTGGATTCTC
mFbxw11-F	TAGCTAGCATGGAGCCCGACTCGGT
mFbxw11-R	TCCAAGCTGCTTTTATCCCAGATCTTG
Keap1-F	GGGCTAGCATGCAGCCCGAACCCAA
Keap1-R	GCAGATCTTGGGCGCGCGGCAG
Lnx1up	GCGCTAGCCATGAACCAACCGGACC
Lnx1down	AAGAGATCTTGGCGGCTGCAGAAACC
LnxSTOPdown	CGAGATCTTAGGCGGCTGCAG
NEDD4-F	AACTCGAGCTACTCCAGGGATTACAAAAG
NEDD4-R	AAGTCGACCTAATCAACTCCATCAAAGCCC
Nb-F	AAAGATCTCGATGGCTCAGGTGCAGCTGC
Nb-R	TTGTCGACTCAGGAGACGGTGACCTGG
ShRING-GBP-F	CGTTCACGGGGAGCGGATGGCATGAAGGCCTGGAAGA
ShRING-R	TCCGCTCCCCGTGAACGGGCAGGTGAC
CIBN-Nb-F	GCGCTAGCATGAATGGAGCTATAGGAG
CIBN-Nb-R	GCGAAGATCTCATGAATATAATCCGTTTTC
CRY2-F	GAGAGCTCCATGAAGATGGACAAAAAGA
CRY2-R	TGGTCGACTTATGCTGCTCCGATCATG
Nb-CIBN-F	GCGAGCTCCATGAATGGAGCTATAGGAG
Nb-CIBN-R	CGAGTCGACTTAATGAATATAATCCGTTTTC
E3-F	CCATGAACCAACCGGACCTTGC
E3-R	GGCGGCTGCAGAAACCT
rEF1aGib-F	GAAAGCCACCGCTAATTCAAAGCAA
E3-Gibson-F	AAGCAACCGGTGCCACCATGAACCAACCGGACCTTGC
E3-CIBN-F	AGAGGTTTCTGCAGCCGCCATGAATGGAGCTATAGGAGGTGACCT
CIBN-HA-R	TCTTATCATGTCTGGCCAGCTAGCTGTACATTACGCGTAGTCTGGCACGTCGTAGGGGT AATGAATATAATCCGTTTTCTCCAATTCC
Nb-Gibson-F	GAAAGCCACCGCTAATTCAAAGCAATCCGGACCGCCATGGCTCAGGTGCAGCTGGTG
Nb-Gibson-R	TCCCACCACCTGAGGAGACGGTGACCTG
linker-CRY2-F	TCACCGTCTCCTCAGGTGGTGGGAGCATGAA
CRY2-myc-R	TGCTCCTAGGCGTACGGGATCCTTAAAGCAGGTCCTCCTCTGAGATCAGCTTCTGCATT GCTGCTCCGATCATGATC
pVitro-ORF2-R	TTGGGGAAACCTGCTCCTAGGCGTACGGGATCC
LnxRING-F	ACCTTAATGGAGGCTCCATGAACCAACCGGA
LnxRING-R	GGCACGTCGTAGGGGTAGGCGGCTGCAGAAAC
HA-R	GGGCGCTAGCTGTACATTACGCGTAGTCTGGCACGTCGTAGGGGT
GID1A-F	TGGTGGGAGCATGCTCGAGGCTGCGAGCGATGAA
GID1A-R	TTAAAGCAGGTCCTCCTCTGAGATCAGCTTCTGCTCACATTCCGCGTTTACAAAC
E3-R2	CTTCATGGAGGCGGCTGCAGAAACCT
GAI-F	AGCCGCCTCCATGAAGAGAGATCATCATCATCATC
GAI-R	GGCACGTCGTAGGGGTAATTAAGGTCGGTGAGCATAG
PLY-F	GGAACCGCCATGCTCGAGACTCAAGACGAATTCACCC

PLY-R	ATGCTGCCTCCTCCGTCGACGTTCATAGCTTCAGTGATCG
ABI-F	GCCGCCTCCATGAAGGTGCCTTTGTATGGTTTTACTTC
ABI-R	TGGTGGTGGTTGATTTGAAGTACCCCTACGACGTGCC
ABI-R2	GGCACGTCGTAGGGGTACTTCAAATCAACCACCACCA
GAI1N-F	CACCGCTAATTCAAAGCAACCGGTGCCACCATGAAGAGAGATC
GAI1N-R	GGAGCCTCCATTAAGGTCGGTGAGCATAG
Vect-GBP-F	GCAATCCGGAACCGCCATGGCTCAGGTGCAGCT
GBP-L-R	GAGCATGCTCCCACCACCTGAGGAGACGGTGACCTG
GBP-L-F	TCCTCAGGTGGTGGGAGCATGCTCGAGGGCGTGCA
FK-myc-V-R	TACGGGATCCTTAAAGCAGGTCCTCCTCTGAGATCAGCTTCTGCATGTCGACTTCCAGTTTTAGAAGCTC
Vect-FK-F	GCAATCCGGAACCGCCATGCTCGAGGGCGTGCA
FK-L-GBP-R	CCTGAGCCATGCTGCCTCCTCCGTCGAC
L-GBP-F	AGGAGGCAGCATGGCTCAGGTGCAGCTG
myc-Vect-R	ACGGGATCCTTAAAGCAGGTCCTCCTCTG
2xlinker-F	TCCTCAGGAGGAGGCTCCGGTGGTGGGAGCATGCTCG
2Xlinker-R	GGAGCCTCCTCCTGAGGAGACGGTGACCTGG
3xlinker-F	CGGTGGCTCTGGAGGAGGCTCCGGTGGTGGGAGCATGCTC
3xlinker-R	CCTCCTCCAGAGCCACCGCCTGAGGAGACGGTGACCTGG
4xlinker-F	GAGCATGCTCCCACCACCGGAGCCTCCTCCAGAGCCACCG
4xlinker-R	GGACCCACCTCCTGAGGAGACGGTGACCTGGG
6xlinker-F	GGCGGCGGCAGTGGCGGAGGATCAGGAGGTGGGTCCGGCGG
6xlinker-R	CCTCCGCCACTGCCGCCGCCTGAGGAGACGGTGACCTGGG
rEF1-F	GAAAGCCACCGCTAATTCAAAGCAA
rEF1-Nb-F	GCAATCCGGAACCGCCATGGCTCAGGTGCAGCT
Nb-R	TGAGGAGACGGTGACCT
Nb-linker-R	CACCGGAGCCTCCTCCGATTGAGGAGACGGTGACCTGG
Nb-Gibson-R	GCTCCCACCACCGGAGCC
FKBP-F	ATGCTCGAGGGCGTGCAGGTGG
FKBP-Gibson-F	CTCCGGTGGTGGGAGCATGCTCGAGGGCGTGCA
FKBP-R	GTCGACTTCCAGTTTTAGAAGCTCCA
FKBP-myc-R	TACGGGATCCTTAAAGCAGGTCCTCCTCTGAGATCAGCTTCTGCATGTCGACTTCCAGTTTTAGAAGCTC
FKBP-Gibson-R	CTGCTCCTAGGCGTACGGGATCCTTAAAGCAGGTCCTCCTCT
mEF1E3-F	AAGCAACCGGTGCCACCATGAACCAACCGGACCTTGC
E3-Gibson-R	CTTCATGCCAGGCGGCTGCAGAAACCT
Frb-Gibson-F	TGCAGCCGCCTGGCATGAAGGCCTGGAA
Frb-HA-R	GCCAGCTAGCTGTACATTACGCGTAGTCTGGCACGTCGTAGGGGTAGAACTGCTTTGAGATTCGTCGG
EcoV-FKBP	CACCGCTAATTCAAAGCAATCCGGAGATATCGGAGGAGGCTCCGGTGGTG
DARPin-F	GCCACCATGGGACCTGGTTCCGATTTGG
DARPin-R	AGCGGCTTTTTGAAGTACCTCG
Nb-F1	ATTCAAAGCAATCCGGAGATGCCACCATGGCTCAGGTGCAGCTGGT
Nb-F2	ATTCAAAGCAATCCGGAGATGCCACCATGGCTCAGGTGCAGCTG
NbLamin-R	CACCGGAGCCTCCTCCGATTGAGGAGACGGTGACCTGG
LaM4-R	CACCGGAGCCTCCTCCGATTGAGCTAACTGTCACCTGAGT

Peft3-GBP-F	TTCAGTTGGGAAACACTTTGCTCTAGAAAAAATGGCTCAAGTTCAGCTCCA
HA-Peft3-R	GCTTGAAAGGATTTTGCATTTATCACTAGTTTACGCGTAATCAGGCACGTC
inter-GBP-F	GGGAAACTGCTGTACCGGTAGAAAAAATGGCTC
inter-GBP-R	CTACCGGTACAGCAGTTTCCCTGAATTAAAATTAG
Peft3-E3-F	TTCAGTTGGGAAACACTTTGCTCTAGAAAAAATGGCGTCTGAAACTAAAGCC
myc-Peft3-R	GCTTGAAAGGATTTTGCATTTATCACTAGTTTAAAGCAAATCTTCCTCTGATAT
LmnA-F	GGGCGATCGCATGGAGACCCCGCTACA
LmnA-R	AGTCGCGGCCGCTTTACATGATGCTGC
MINexternal-F	CAGAAGCCAGCCTTTCGCTCCC
MINexternal-R	CCTGCGAGCCTCGGTTTTCTCC
CENPA-seq-F	TCTACGTAAGGGGCGTTCCA
CENPA-seq-R	GGATCGGGACTCGGGAGAT
attL_F	CCGGCTTGTCGACGACG
MIN-tag DNA donor oligo	GGCCGGGGGTCGGGGTCGGGCTCGGGCTGCGCCTCCTCGGGGCCTCGGGCTTTCGGC TCCGGCGGCGCGGGCCGGTTTGTACCGTACACCACTGAGACCGCGGTGGTTGACCAGA CAAACCGAGCATGACACGCCGCAGAGGGTGCTGGCGCCCGGTCCCACGGCTCCTGCTC GGGCTGCCGGGTCCGGGAGCGAAAGGC

Supplementary Table 2. Plasmids used in this study.

name	prokaryotic resistance	internal number	used in	reference
pFbx1A-GBP1-IR	Kana	pc4209	SF.1	this study
pFbx1B-GBP1-IR	Kana	pc4211	SF.1	this study
pKeap1-GBP1-IR	Kana	pc4212	SF.1	this study
pLnx1-GBP1-IR	Kana	pc4213	SF.1	this study
pGBP1-Nedd4-IR	Kana	pc4214	SF.1	this study
pGBP1-IR	Kana	pc4215	SF.1	this study
pRINGsh-GBP1-IR	Kana	pc4216	SF.1	this study
pLL7.0: Venus-iLID-CAAX	Amp	Addgene Plasmid #60411	SF.2	Guntas et al. 2016
pLL7.0: hITSN1(1159-1509)-tgRFPt-SSPB WT	Amp	Addgene Plasmid #60419	SF.2	Guntas et al. 2016
pCRY2PHR-mCherryN1	Kana	Addgene Plasmid #26866	SF.2, SF.5	Kennedy et al. 2010
pCIBN(deltaNLS)-pmGFP	Kana	Addgene Plasmid #26867	SF.2, SF.5	Kennedy et al. 2010
pNLSGFP-iRFP670-PCNA	Kana	pc4222	SF.2	this study
pCIBN-GBP1-IR	Kana	pc4223	SF.2, SF.3	this study
pGBP1-CIBN-IR	Kana	pc4224	SF.3	this study
pRING-PHR-IR	Kana	pc4225	SF.3	this study
pGBP1-PHR-IR	Kana	pc4226	SF.3	this study
pCIBN-RING-IR	Kana	pc4227	SF.3	this study
pVitro-LiPD-GBP1-IR	Hyg	pc4228	SF.3	this study
pVitro-LiPD-GBP2-IR	Hyg	pc4229	SF.3	this study
pVitro-LiPD-GBP4-IR	Hyg	pc4231	SF.3	this study
Piggybac-LiPD-GBP1-IR	Amp/Hyg	pc4232	Fig.1, 4 , SF.5, 11	this study
pSpCas9(BB)-2A-GFP (PX458)	Amp	Addgene Plasmid #48138	SF.4	Ran et al., 2013
pCAG-NLS-HA-Bxb1	Amp	Addgene Plasmid #51271	SF.4	Hermann et al. 2014
pattb-EGFP-CENPA	Kana	pc4233	SF.4, 5, 10	this study
pCAG-EGFP-Cxxc4	Amp	pc2311	Fig. 1, 4; SF.11	Liu et al. 2013
piggyBacRING-ABI1/PLY1-GBP5-IR	Amp/Hyg	pc4235	SF.6	this study

piggyBacRING-GAI/GBP5-GID1A-IR	Amp/Hyg	pc4236	SF.6	this study
piggyBacGAI-RING/GBP5-GID1A	Amp/Hyg	pc4237	SF.6	this study
pRING-FRB-HA /FKBP-GBP5-myc	Hyg	pc4238	SF.6	this study
pVitro-DiPD0L	Hyg	pc4242	SF.6	this study
pVitro-DiPD2L	Hyg	pc4243	SF.6	this study
pVitro-DiPD3L	Hyg	pc4244	SF.6	this study
pVitro-DiPD4L	Hyg	pc4245	SF.6	this study
pVitro-DiPD6L	Hyg	pc4246	SF.6	this study
pVitro-DiPD-GBP5-IR	Hyg	pc4247	Fig.4, SF.11	this study
Piggybac-DiPD-GBP5-IR	Amp/Hyg	pc4248	Fig.4, SF.6	this study
pVitro-E3FRB-GBP5	Hyg	pc4234	SF.6	this study
pCAG-EGFP-Lbr-IB	Amp	pc2631	SF.7	this study
pEGFP-N1_hTrex1-WT	Kana	-	SF.7	Wolf et al. 2016
pCAG-EGFP-IDH1-IB	Amp	pc3492	SF.7	this study
pCAG-EGFP-Ahcy-IB	Amp	pc3688	SF.7	this study
pCAG-EGFP-Man1-IB	Amp	pc3081	SF.7	this study
pVitro-DiPD base	Hyg	pc4249	SF.7	this study
pVitro-DiPD-DARPin-IR	Hyg	pc4251	SF.7	this study
pVitro-DiPD-LaM4	Hyg	pc4252	SF.7	this study
pPGK-mCh-LaminA	Amp	pc4253	SF.7	this study
Piggybac-DiPD-aLamin-IR	Amp/Hyg	pc4254	Fig.4, SF.9, 10,11	this study
Piggybac-DiPD-aPCNA-IR	Amp/Hyg	pc4255	Fig.3,4; SF.9,10	this study
pGFP-LIG1	Kana	pc613	Fig.3	Mortusewicz et al. 2006
$P_{eft\text{-}3}$*ring::frb::ha::gpd-2/gpd-3::gbp5::fkbp::myc*_3'UTR	Amp	pc4257	Fig.5	this study
$P_{ced\text{-}3}$*ced-3::gfp*	Amp	-	Fig.5	this study
$P_{eft\text{-}3}$*mkate2*	Amp	-	Fig.5	this study

Kana: kanamycin; Amp: ampicillin; Hyg: hygromycin B; SF: Supplementary Figure

Supplementary Table 3. Cell lines and worm strains used in this study.

**cell line**	**species**	**cell type**	**reference**
HeLa Kyoto	human	cervical adenocarcinoma cells	RRID:CVCL_1922
MEF	mouse	embryonic fibroblast cells	ATCC
HeLa MIN-CENPA	human	cervical adenocarcinoma cells	this study
HeLa GFP-CENPA	human	cervical adenocarcinoma cells	this study
HeLa GFP-CENPA LiPD	human	cervical adenocarcinoma cells	this study
HeLa GFP-CENPA DiPD	human	cervical adenocarcinoma cells	this study
MEF GFP-LaminA	mouse	embryonic fibroblast cells	Chiu et al. 2016
MEF GFP-LaminA DiPD	mouse	embryonic fibroblast cells	this study
MEF mCh-LaminA	mouse	embryonic fibroblast cells	this study
HeLa GFP-PCNA	human	cervical adenocarcinoma cells	Chagin et al. 2016
HeLa GFP-CXXC4	human	cervical adenocarcinoma cells	this study
HeLa GFP-CXXC4 LiPD	human	cervical adenocarcinoma cells	this study
HeLa DiPD	human	cervical adenocarcinoma cells	this study
BHK	hamster	kidney cells	Tsukamoto et al. 2000

**worm strain**	**description**	**reference**
LGIV *ced-3* (*n717*)	*ced-3(lf)* loss of function	Ellis and Horvitz. 1986
LGV *bcSi36*<br>*(derived from ced-3 (n717))*<br>($P_{ced\text{-}3}$*ced-3::gfp*)	*ced-3(lf)* loss of function<br>*ced-3::gfp* transgene	this study
bcEx1328 (derived from bcSi36)<br>($P_{eft\text{-}3}$*ring::frb::ha::gpd-2/gpd-3::gbp5::fkbp::myc*_3'UTR; $P_{eft\text{-}3}$*mkate2*)	*ced-3(lf)* loss of function<br>*ced-3::gfp* transgene<br>and DiPD system on<br>extrachromosomal array	this study
