# Supplementary material for: Tunable light and drug induced depletion of target proteins
Source: Nat Commun. 2020 Jan 16;11:304. doi: 10.1038/s41467-019-14160-8 (PMC6965615; doi:10.1038/s41467-019-14160-8)
Supplement: Supplementary file 2 — Description of Additional Supplementary Files [file 41467_2019_14160_MOESM2_ESM.pdf]

Description of Additional Supplementary Files

File name: Supplementary Movie 1

Description: Light induced association of tagged protein with an intracellular target and subsequent dissociation. Upon light induction the PHR-mCh heterodimerized with CIBN fused to a GFP binding nanobody (GBP1-CIBN) leading to visible recruitment at nuclear GFP-PCNA foci. Over the following minutes in the dark, a gradual dissociation of the PHR-mCh fusion from nuclear PCNA foci can be observed (s. detailed description in Supplementary Fig. 2c,d). Time is shown as mm:ss.

File name: Supplementary Movie 2

Description: Light induced depletion of GFP-PCNA. Cells expressing the LiPD construct can be identified by the co-expressed DsRed. After light induction, GFP-PCNA was depleted from these cells as described in more detail in Supplementary Fig. 3d. Time is shown as hh:mm.

File name: Supplementary Movie 3

Description: Light induced depletion of GFP-CXXC4 in cells stably expressing the LiPD. Cells expressing the LiPD construct can be identified by the co-expressed DsRed. After light induction, GFP-CXXC4 was depleted from these cells as described in more detail in Fig. 1b. As internal control, cells without LiPD (green only) were mixed-in. Time is shown as hh:mm.

File name: Supplementary Movie 4

Description: Light induced depletion of CENPA. A GFP-CENPA fusion was introduced at the endogenous locus via CRISPR/Cas9 mediated recombination. Light induced depletion of GFP-CENPA could be observed in cells stably expressing the LiPD construct (identified by DsRed, in red), but not in neighboring control cells without the LiPD system. A detailed description can be found in Supplementary Fig. 4c. Time is shown as hh:mm.

File name: Supplementary Movie 5

Description: Induced depletion of GFP-LMNA with the DiPD system in mouse embryonic fibroblast cells (MEFs). GFP- LMNA was depleted in cells expressing the DiPD (identified by the co-expressed DsRed, in red) after rapamycin induction but not in control cells without the DiPD (green only). This supplement is the full movie corresponding to the snapshots shown in Fig. 2c. Time is shown as hh:mm.

File name: Supplementary Movie 6

Description: Recruitment of DNA ligase I (LIG1) to the DNA damage repair (DDR) site in the presence or absence of PCNA. Endogenous PCNA was depleted in cells with the DiPD system (DsRed positive, in red) but not in adjacent wt cells lacking the DiPD system (DsRed negative). DNA damage was induced by laser in both types of cells (damaged sites are marked by circles). In wt cells rapid recruitment of GFP-LIG1 to the DDR site (filled arrowhead) could be observed, while little to no recruitment of GFP-LIG1 to the DDR site (open arrowhead) was monitored in cells with PCNA depletion. A detailed description can be found in Fig. 3b,c. Time is shown as mm:ss.
