# Supplementary material for: Tunable light and drug induced depletion of target proteins
Source: Nat Commun. 2020 Jan 16;11:304. doi: 10.1038/s41467-019-14160-8 (PMC6965615; doi:10.1038/s41467-019-14160-8)
Supplement: Supplementary file 9 — Reporting Summary [file 41467_2019_14160_MOESM9_ESM.pdf]

nature research

Corresponding author(s): Heinrich Leonhardt

Last updated by author(s): Nov 23, 2019

Reporting Summary

Nature Research wishes to improve the reproducibility of the work that we publish. This form provides structure for consistency and transparency in reporting. For further information on Nature Research policies, see Authors & Referees and the Editorial Policy Checklist .

Statistics

For all statistical analyses, confirm that the following items are present in the figure legend, table legend, main text, or Methods section.

n/a	Confirmed	
☐	☒	The exact sample size (n) for each experimental group/condition, given as a discrete number and unit of measurement
☐	☒	A statement on whether measurements were taken from distinct samples or whether the same sample was measured repeatedly
☒	☐	The statistical test(s) used AND whether they are one- or two-sided<br>*Only common tests should be described solely by name; describe more complex techniques in the Methods section.*
☒	☐	A description of all covariates tested
☒	☐	A description of any assumptions or corrections, such as tests of normality and adjustment for multiple comparisons
☐	☒	A full description of the statistical parameters including central tendency (e.g. means) or other basic estimates (e.g. regression coefficient) AND variation (e.g. standard deviation) or associated estimates of uncertainty (e.g. confidence intervals)
☒	☐	For null hypothesis testing, the test statistic (e.g. F, t, r) with confidence intervals, effect sizes, degrees of freedom and P value noted<br>*Give P values as exact values whenever suitable.*
☒	☐	For Bayesian analysis, information on the choice of priors and Markov chain Monte Carlo settings
☒	☐	For hierarchical and complex designs, identification of the appropriate level for tests and full reporting of outcomes
☒	☐	Estimates of effect sizes (e.g. Cohen's d, Pearson's r), indicating how they were calculated

Our web collection on statistics for biologists contains articles on many of the points above.

Software and code

Policy information about availability of computer code

Data collection	ll software used in this study for data collection are commercially available.<br>Leica SP5 confocal microscope was controlled with LAS AF (Version 2.0.2, Leica Microsystems)<br>Nikon Ti2 microscope was controlled with NIS-Elements Ar(Version 5.02.00, Nikon)<br>UltraVIEW VoX microscope was controlled with Volocity (Version 6.1.2, PerkinElmer)<br>Infinite M1000 plate reader was controlled with i-control (Version 1.10, Tecan)<br>Operetta HCS microscope was controlled with Harmony (Version 3.5, PerkinElmer)<br>FACS Aria II was controlled with BD FACSDiva (Version 5.0.3, BD Biosciences)
Data analysis	All software used in this study for data analysis are either commercially available or open source.<br>ImageJ (Fiji, version 1.52h)<br>Excel (Office 2007, Microsoft)<br>R (RStudio version 1.1.456, RStudio Team)<br>Harmony (Version 3.5, PerkinElmer)<br>FlowJo (Version 9.6.2, FlowJo)<br>Clustal Omega (Online tool, Uniprot website)<br>Figures were organized with Adobe Illustrator (Version CS5.1, Adobe)<br>Movies were converted with Adobe Media Encoder (Version CS5.5, Adobe)

For manuscripts utilizing custom algorithms or software that are central to the research but not yet described in published literature, software must be made available to editors/reviewers. We strongly encourage code deposition in a community repository (e.g. GitHub). See the Nature Research guidelines for submitting code & software for further information.

Data

Policy information about availability of data

All manuscripts must include a data availability statement. This statement should provide the following information, where applicable:

- Accession codes, unique identifiers, or web links for publicly available datasets
- A list of figures that have associated raw data
- A description of any restrictions on data availability

The data that support the findings of this study are available upon reasonable request.

Field-specific reporting

Please select the one below that is the best fit for your research. If you are not sure, read the appropriate sections before making your selection.

☒ Life sciences ☐ Behavioural & social sciences ☐ Ecological, evolutionary & environmental sciences

For a reference copy of the document with all sections, see nature.com/documents/nr-reporting-summary-flat.pdf

Life sciences study design

All studies must disclose on these points even when the disclosure is negative.

Sample size	No statistic methods were used to predetermine the sample size as the variation between the samples was minimal.<br>For confocal microscopic experiments, analyzed sample size was typically between 15 - 30, as indicated in the text.<br>For high content image analysis, large image fields (121 fields with several thousands cells) were analyzed.<br>In FACS analyses, sets of 10,000 cells were directly compared.<br>For cell population experiments with the fluorescence plate reader, cell numbers were chosen according to the detection limit of the instrument.
Data exclusions	For live cell imaging experiments, cells which moved out of the field of view during long term imaging were not included for data collecting.<br>In the multiple protein depletion experiments with transient triple transfections, cells with only single or double transfection were excluded.
Replication	All microscopic experiments were performed multiple times. FACS analyses and cell population assays were performed as triplicates. All experimental replicates were successful.
Randomization	For LiPD and DiPD experiments, cells belong to experimental and control groups were mixed and received the same physic or chemical treatment.<br>For both fixed and live cell experiments, image fields were randomly chosen for confocal imaging. For transient transfection experiments, transfected cells were randomly chosen for imaging.
Blinding	The investigators were not blinded during data collection.

Reporting for specific materials, systems and methods

We require information from authors about some types of materials, experimental systems and methods used in many studies. Here, indicate whether each material, system or method listed is relevant to your study. If you are not sure if a list item applies to your research, read the appropriate section before selecting a response.

Materials & experimental systems

n/a	Involved in the study	
☐	☒	Antibodies
☐	☒	Eukaryotic cell lines
☒	☐	Palaeontology
☐	☒	Animals and other organisms
☒	☐	Human research participants
☒	☐	Clinical data

Methods

n/a	Involved in the study	
☒	☐	ChIP-seq
☐	☒	Flow cytometry
☒	☐	MRI-based neuroimaging

Antibodies

Antibodies used	Mouse anti- c-myc (9E10, from Thermo Scientific)<br>Rat anti- HA (3F10, from Sigma Aldrich)<br>Rabbit anti- LmnA/C (EP4520, from EMD Millipore)<br>Rat anti- PCNA (16D10), generated by our lab and published in [Hybridoma, 27(5):337-343].

	Alexa Fluor 405 labeled goat anti- mouse (A31553, Life Technology)<br>Alexa Fluor 594 labeled goat anti- rabbit (A11034, Life Technology)<br>Alexa Fluor 647 labeled goat anti- rat (A21247, Life Technology)<br>Alexa Fluor 594 labeled goat anti- rat (A11007, Life Technology)<br>Alexa Fluor 488 labeled donkey anti- rat (A21208, Life Technology)<br>Alexa Fluor 647 labeled donkey anti- mouse (A31571, Life Technology)
Validation	Commercial antibodies were purchased as validated by the manufacturers. The antibody for PCNA has been extensively tested and used in the past [Nucleic Acids Res., 41(9):4860–4876; Biophys J., 109(8):1551–1564; Nat. Commun., 7, 11231]. The anti- c-myc antibody and anti- HA antibody were also tested by immunostaining in this study.

Eukaryotic cell lines

Policy information about cell lines

Cell line source(s)	All cell lines used in this study are listed in Supplementary Table 1.<br>BHK(#3, with lacO array) cells were from [Nat. Cell Biol., 2(12):871-878].<br>MEF cells were from [Hum. Mol. Genet., 26(8):1522–1534].<br>MEF GFP-LaminA cells were from (Sci. Rep., 6, 25019).<br>HeLa GFP-PCNA cells were from (Nat. Commun., 7, 11231).<br>HeLa Kyoto (RRID: CVCL_1922)<br>All other LiPD or DiPD cell lines generated in this study were derived from HeLa Kyoto or MEF cells.
Authentication	No cell lines used were authenticated.
Mycoplasma contamination	All cell lines used in this study tested negative for mycoplasma contamination by PCR.
Commonly misidentified lines<br>(See ICLAC register)	No commonly misidentified lines (ICLAC, version 9) were used in this study.

Animals and other organisms

Policy information about studies involving animals; ARRIVE guidelines recommended for reporting animal research

Laboratory animals	C. elegans strains used are described in the Methods section.
Wild animals	This study did not use any wild animals.
Field-collected samples	This study did not involve any samples collected from the field.
Ethics oversight	*Identify the organization(s) that approved or provided guidance on the study protocol, OR state that no ethical approval or guidance was required and explain why not.*

Note that full information on the approval of the study protocol must also be provided in the manuscript.

Flow Cytometry

Plots

Confirm that:

- [x] The axis labels state the marker and fluorochrome used (e.g. CD4-FITC).
- [x] The axis scales are clearly visible. Include numbers along axes only for bottom left plot of group (a 'group' is an analysis of identical markers).
- [x] All plots are contour plots with outliers or pseudocolor plots.
- [ ] A numerical value for number of cells or percentage (with statistics) is provided.

Methodology

Sample preparation	Cultured cells were transfected with different E3 constructs and then trypsinized for analysis.
Instrument	BD FACSAria II, special order system.
Software	BD FACSDiva (Version 5.0.3, BD Biosciences), FlowJo (Version 9.6.2, FlowJo).
Cell population abundance	For each of the tested groups, 10,000 transfected cells (DsRed positive) were analyzed. The transfection efficiency varied between 14% to 30%. Biological triplicates were performed.

Gating strategy

Gating of fluorescent positive cells were according to untransfected HeLa cells.

☐ Tick this box to confirm that a figure exemplifying the gating strategy is provided in the Supplementary Information.
